# Marine environmental DNA biomonitoring reveals seasonal patterns in biodiversity and identifies ecosystem responses to anomalous climatic events

**DOI:** 10.1371/journal.pgen.1007943

**Published:** 2019-02-08

**Authors:** Tina E. Berry, Benjamin J. Saunders, Megan L. Coghlan, Michael Stat, Simon Jarman, Anthony J. Richardson, Claire H. Davies, Oliver Berry, Euan S. Harvey, Michael Bunce

**Affiliations:** 1 Trace and Environmental DNA (TrEnD) Laboratory, School of Molecular and Life Sciences, Curtin University, Bentley, Western Australia; 2 Fish Ecology Laboratory, School Molecular and Life Sciences, Curtin University, Bentley, Western Australia; 3 School of Biological Sciences, Macquarie University, Sydney, Australia; 4 CSIRO Environomics Future Science Platform, Indian Ocean Marine Research Centre, The University of Western Australia, Perth, Western Australia; 5 Centre for Applications in Natural Resource Mathematics, School of Mathematics and Physics, The University of Queensland, St Lucia, Queensland, Australia; 6 CSIRO Oceans and Atmosphere, Queensland Biosciences Precinct, St Lucia, Queensland, Australia; 7 CSIRO Oceans and Atmosphere, Castray Esplanade, Hobart, Tasmania, Australia; University of Copenhagen, DENMARK

## Abstract

Marine ecosystems are changing rapidly as the oceans warm and become more acidic. The physical factors and the changes to ocean chemistry that they drive can all be measured with great precision. Changes in the biological composition of communities in different ocean regions are far more challenging to measure because most biological monitoring methods focus on a limited taxonomic or size range. Environmental DNA (eDNA) analysis has the potential to solve this problem in biological oceanography, as it is capable of identifying a huge phylogenetic range of organisms to species level. Here we develop and apply a novel multi-gene molecular toolkit to eDNA isolated from bulk plankton samples collected over a five-year period from a single site. This temporal scale and level of detail is unprecedented in eDNA studies. We identified consistent seasonal assemblages of zooplankton species, which demonstrates the ability of our toolkit to audit community composition. We were also able to detect clear departures from the regular seasonal patterns that occurred during an extreme marine heatwave. The integration of eDNA analyses with existing biotic and abiotic surveys delivers a powerful new long-term approach to monitoring the health of our world’s oceans in the context of a rapidly changing climate.

## Author summary

All environments contain genetic remnants of the life they contain and support. For example, samples collected from the ocean contain biological material such as microscopic organisms, shed cells, excrement and saliva—the DNA from which reveals the surrounding marine biodiversity. Environmental DNA (eDNA) approaches have the ability to identify marine species that are notoriously difficult to identify using morphology alone. Here we develop, and apply, a DNA ‘toolkit’ to five years of samples collected from a single site in the Indian ocean. It is rare to find a temporal series of samples of this duration that are also suitable for DNA analysis. We show that eDNA techniques have the capacity to monitor ocean biology in fine detail. We demonstrate how the biological communities of plankton and fish respond to normal seasonal changes and, more importantly, to that of an uncharacteristic heatwave. The methods embodied in this paper are applicable to marine studies across the globe and, as such, pave the way for the design of long-term monitoring programs using eDNA.

## Introduction

Changes in ocean temperatures, chemistry and currents are occurring faster now than at any time in human history [1, 2]. These changes will certainly impact the productivity in marine environments that is critical for social and economic wellbeing [3]. These impacts have driven the expansion of global efforts to monitor marine biota and track ecosystem health [1, 4, 5]. Abiotic environmental data are already collected by various methods across all oceans [4, 6], but thorough sampling of marine biota is far more restricted and challenging [5]. Robust biomonitoring programs that link biological changes to the physio-chemical state of the oceans will help to identify ecological trends and predicting future trajectories.

Since 1931, the biomass and morphological species in zooplankton communities have been used extensively for oceanic biomonitoring [7]. Zooplankton are the trophic link between phytoplankton and larger predators [8]. These highly diverse communities have been described as ‘beacons of change’ [9], as their community composition is known to respond to fluctuations in both abiotic and biotic factors [5, 9, 10]. Most zooplankton are ectothermic, so they are sensitive to temperature changes that affect their physical activity and physiology [9]. Many species are also fast growing and short-lived and so communities typically respond rapidly to changes in environmental conditions [5, 9–11].

The importance of extended temporal sampling to describe changes within planktonic communities has long been recognised [1, 4, 5, 12–14]. A long-term analysis has the ability to define baselines and understand what is ‘normal’ for a community [4] and provides a mechanism to gauge ecosystem health [11]. There are several extended studies targeting zooplankton [1, 4, 5, 12, 14–17], yet these typically focus on a narrow range of taxa [1, 11, 13, 18–20].

Morphological identification of zooplankton is time consuming and expensive [4, 21]. Samples must be in good physical condition, particularly for taxonomic identifications reliant on the presence of fragile appendages. This problem is worst for easily damaged, soft-bodied phyla such as Cnidaria and Ctenophora [22]. Many marine animals, including fish and larger crustaceans, have a larval planktonic phase, and identification of larvae to species is difficult or impossible, even for skilled taxonomists [21, 23, 24]. Morphological studies tend to overestimate the relative abundance of those taxa that are readily identified, but overlook a significant fraction of marine animal groups. Consequently there is growing recognition that morphology by itself will struggle to meet with the increasing need for holistic marine biomonitoring in conservation and management decisions [4, 25].

Environmental DNA (eDNA) is transforming our ability to study marine biodiversity. Recent metabarcoding studies on eDNA extracted from water [26–28], sediment [29], scat [25, 30–33] and plankton [21, 34] demonstrate its capacity to profile a vast range of biota. While these studies focus strongly on spatial and community differences, the ability for eDNA to act as a long-term temporal biomonitoring tool is unexplored.

**Fig 1:**
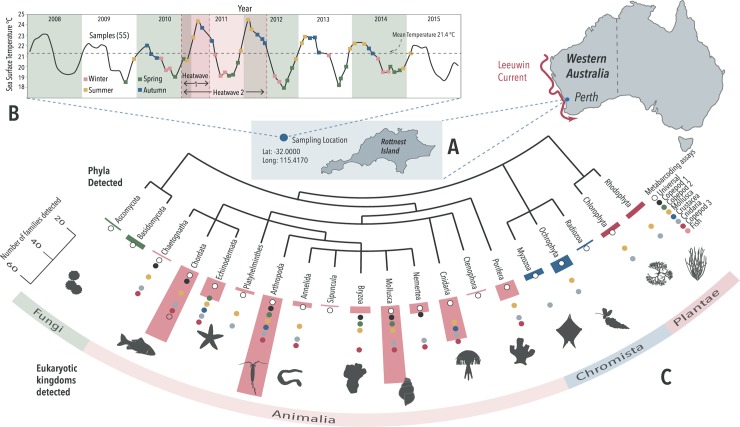
Extent of marine taxa revealed by eDNA from Rottnest Island (A). 55 monthly plankton samples taken across five years (2009–2015) and an extreme heatwave event (B), which yielded 245 families of eukaryotic zooplankton across 20 phyla (C).

Environmental DNA is defined in Taberlet *et al* [35] as a “*complex mixture of genomic DNA from many different organisms found in an environmental sample… [including] material resulting from filtering air or water*, *from sifting sediments*, *or from bulk samples*”. Here, due to historic sampling, we analyse eDNA purified from bulk zooplankton samples systematically collected monthly over five-years from a single ecologically significant site at Rottnest Island, Western Australia [36] (Fig 1A). This temporal window of sampling includes a “marine heatwave” anomaly that had significant impacts on the south Western Australian marine ecosystem [37–39]. We test the capacity for eDNA metabarcoding to track biotic shifts, examine how eDNA signatures relate to abiotic variables, and lastly outline the value and practical implementation of multi-year eDNA programs

Shotgun DNA sequencing has been used for eDNA community analysis [40] but it is cost prohibitive and dominated by prokaryotic taxa [28]. Single marker metabarcoding approaches have proven useful for biological monitoring, but their taxonomic focus has to be narrow because each assay is by definition limited in scope. Even supposedly “universal” DNA metabarcoding assays have proven inadequate to identify a comprehensive range of target taxa in our global oceans [28]. To address the challenge of pinpointing a range of metazoan taxa, we developed a novel multi-gene (COI, 16S & 18S) metabarcoding ‘toolkit’ capable of working with both degraded and intact eDNA, and able to identify a wide variety of taxa found within zooplankton communities. We used three existing metabarcoding assays and designed five more (S1 Table) to target a range of crustaceans, molluscs, fish and cnidarians known to be present at the reference site [41]—a site that has been monitored using a variety of methods since 1951 [6].

## Results and discussion

Overall, while the majority of the eDNA extracted during this study originates from the plankton sampled (including larva and eggs), a small amount (impossible to quantify) would derive from sloughed cells or faecal material from larger organisms. From this total DNA more than four hundred distinct eukaryotic taxa were identified in this five-year study. These taxa were identified from more than nine million metabarcode sequences clustered into four thousand unique high abundance groups. Across all time points and assays, a total of 20 eukaryotic phyla were detected containing 245 families (Fig 1; S2–S7 Tables). Fig 1B also depicts the surface temperature and chronology of collection at the monitoring site. Most detections (70%) were within Arthropoda (including 62 families) and, of these, 87% were from Hexanauplia (including 24 families), the class that contains all copepods. The metabarcoding method employed here identified some of the gelatinous and larval zooplankton such as over 15 genera of hydrozoa and 50 genera of actinopterygii, many to species level. In practice, all assays, with the exception of the Fish assay, detected an extremely broad range of taxa. The Copepod 3 assay alone was responsible for over 1100 assignments across ten Animalia phyla; almost a quarter of all detections. It is, however, the integration of all assays that has revealed some of the breadth of biodiversity within this ecosystem over the five-year period. Had the study been limited to the 18S Universal assay, fewer than 70 assignments would have been made.

While Fig 1C showcases the taxa that our assays detected, more than 40% of the DNA sequences could not be reasonably assigned within a taxonomic framework. As a consequence of this problem, we applied a taxonomy-independent approach so that the analyses were not biased by the limitations of reference databases or the accuracy of the underpinning taxonomy. Operational Taxonomic Units (OTUs) enabled a more comprehensive exploration of the correlations between biotic and abiotic change over time.

### Seasonal & annual patterns

Biological monitoring at a single point in time is typically inadequate to describe total biodiversity or to explore changes in diversity over time. Collecting multiple time-stamped samples reveals greater total (gamma) biodiversity and allows measurement of beta diversity as a temporal change. For each assay, OTU biodiversity analysis involved both counting of the number of discrete OTUs—a measure hereafter referred to as “Richness”—and the presence/absence composition of the OTUs—referred to as “Assemblage”. OTUs from each assay were examined independently so that comparisons were all made within the same experimental frameworks.

There are varying approaches for presenting eDNA metabarcoding data in terms of Assemblage and Richness. Some authors rarefy their data to normalise results for differing sequencing depth among libraries. We made the decision not to do this because sequence number and OTU accumulation curves had plateaued for each sample indicating that we had sampled the majority of the OTUs in each case (For example; S1 Fig), Pearson’s correlation tests showed there was no evidence to suggest a significant correlation between the number of sequences (i.e. sequencing depth) and the number of OTUs obtained for the 18S and 16S assays (S2 Fig). However, sequencing depth and number of OTUs (Richness) were moderately correlated (R^2^<0.522) for the COI assays (S2 Fig). Nevertheless, as sequencing depth variation is spread evenly across the samples (S2 Fig), we consider it unlikely that Richness or Assemblage estimates are compromised by this data treatment.

Our initial analyses of eDNA (Table 1) demonstrated strong seasonality in the Assemblage from those assays that predominately detect meroplankton, including fish, molluscs and cnidarians. This seasonality was not reflected in Richness, with the exception of the Fish assay. A pairwise analysis between seasons (S8 Table) indicated that the most consistent differences in Assemblage were detected between summer:winter, followed by spring:winter and spring:autumn. The least significant Assemblage changes were identified by the assays that predominantly detect holoplankton e.g. the Copepod assays. These detected no significant changes (after *post-hoc* correction) between winter:autumn, and summer:spring. These results provide a detailed example for multi-year marine biodiversity surveys based on eDNA.

**Table 1:** Significance of changes to the Operational Taxonomic Unit (OTU) Richness (a count of the number of OTUs in each sample) & Assemblage (the OTUs making up each sample) during different time periods within the five-year eDNA data including F statistics (F)—PERMANOVA+ [42].

Assay (Number of individual OTUs)	OTU diversity test	Main tests 2010–2014			Main tests Before, During and After	
		Month df (30,51)	Season df (15,51)	Year df (4,51)	Heatwave 1 Nov 2010 –April 2011; df (2,54)	Heatwave 2 Nov 2010 –May2012; df (2,54)
**Cnidaria**	Richness	- F = 2.03	- F = 0.93	- F = 1.16	- F = 1.03	- F = 0.16
(246 OTUs)	Assemblage	- F = 0.91	** F = 1.38	** F = 1.58	*** F = 2.80	*** F = 2.84
**Copepod 1**	Richness	- F = 4.89	- F = 1.21	** F = 4.63	** F = 6.60	* F = 4.16
(171 OTUs)	Assemblage	* F = 2.15	* F = 1.41	*** F = 1.99	*** F = 3.28	*** F = 3.64
**Copepod 2**	Richness	- F = 2.76	- F = 0.71	- F = 1.50	- F = 1.67	- F = 0.34
(124 OTUs)	Assemblage	- F = 0.10	- F = 0.22	- F = 1.48	- F = 1.48	* F = 2.01
**Copepod 3**	Richness	- F = 2.76	- F = 0.71	- F = 1.50	- F = 1.67	- F = 0.34
(342 OTUs)	Assemblage	**F = 2.31	*** F = 1.48	- F = 1.35	**F = 1.94	**F = 2.28
**Crustacea**	Richness	- F = 0.55	- F = 1.26	- F = 1.96	- F = 2.41	* F = 3.59
(132 OTUs)	Assemblage	- F = 0.86	* F = 1.29	- F = 1.15	- F = 1.08	- F = 1.32
**Fish**	Richness	- F = 2.38	** F = 3.49	- F = 0.58	- F = 0.09	- F = 0.21
(87 OTUs)	Assemblage	*F = 2.88	*** F = 1.77	- F = 0.79	- F = 1.18	- F = 0.85
**Mollusca**	Richness	- F = 2.21	- F = 1.27	- F = 0.32	- F = 0.57	- F = 0.47
(345 OTUs)	Assemblage	- F = 1.00	*** F = 1.65	* F = 1.39	*** F = 2.41	*** F = 2.29
**Universal**	Richness	- F = 0.52	- F = 0.96	- F = 2.02	- F = 0.40	- F = 1.78
(97 OTUs)	Assemblage	- F = 0.96	* F = 1.34	- F = 1.45	** F = 2.08	*** F = 2.45

Where ***is P ≤ 0.001, **is P ≤ 0.01, *is P≤ 0.05 & – is no significant change

**Fig 2:**
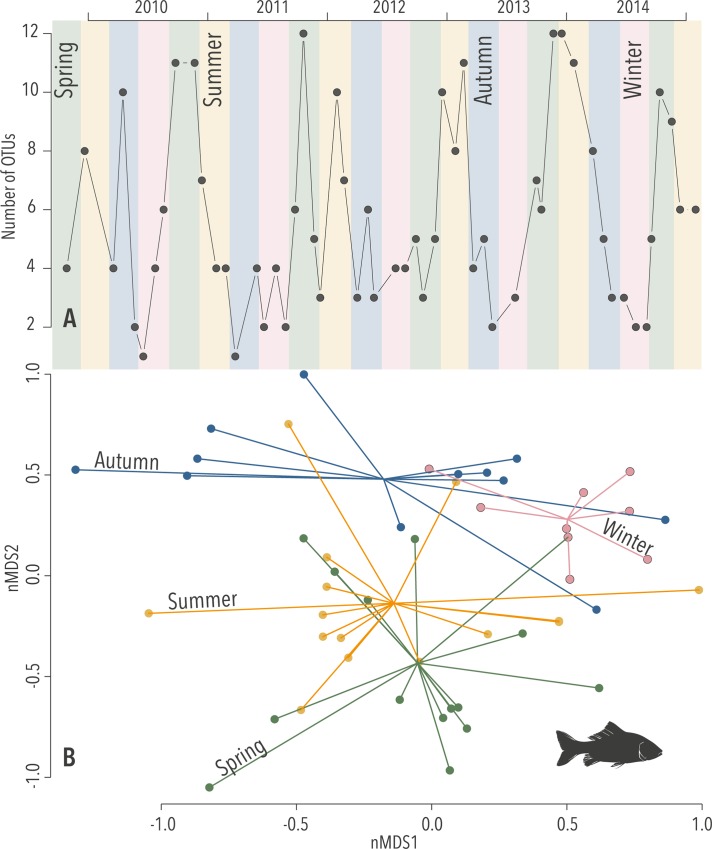
Seasonality in eDNA revealed by the Fish assay: (A) Number of Operational Taxonomic Units (OTUs) at each time point (Richness; *p* < 0.001) and (B) Diversity of OTUs as exhibited by a non-parametric multivariate analysis (Assemblage; *k* = 3, stress = 0.15, *p* < 0.001), the coloured lines extrude from the centroids of each season towards the variation of Assemblage in each sample.

The Fish assay revealed strong seasonality in both Richness and Assemblage (Fig 2). A pairwise analysis showed significant changes between all seasons for the Assemblage as well as Richness (S8 Table), the two exceptions were for Richness between the adjacent seasons summer:spring and winter:autumn. Most fish are only present in the zooplankton community after broadcast spawning their eggs or during their pelagic larval phase, so these seasonal changes make biological sense [24]. Seasonal fluctuations have been previously observed in fish using eDNA extracted from water [43, 44]. However, these studies were limited to durations of six and twelve months respectively. The current study provides additional and enduring evidence for the ability of eDNA to detect of seasonality over an extended period (5 years) and further incorporates a much broader range of biodiversity.

OTUs that characterise particular time periods were identified by *indval* analysis [45]. The strong seasonality in the Fish OTUs suggests that they might be driving significant differences identified in the seasonal *indval* analyses across all assays (S9 Table), but this was not the case. Spring was characterised by a significant indicator matched to Labridae (a speciose fish family), but *Calcinus dapsiles* (a hermit crab) and *Evadne spinifera* (a water flea) were the summer’s four top indicators. *Calcinus dapsiles* are only planktonic as larvae and only present seasonally, but *E*. *spinifera* is part of the plankton for its entire life.

*Flaccisagitta enflata* (a chaetognath or predatory arrow worm) and the copepods *Farranula gibbula* and *Centropages orsinii* were the most significant indicators for autumn. The copepods, *Canthocalanus pauper* and *Centropages furcatus* were found in winter. The genetic assignment of *C*. *orsinii* and *C*. *furcatus* are of interest as they are typically tropical species found in the Indian Ocean [46] indicating that they are likely to have been swept south by the warm water Leeuwin current (Fig 1A) in each year [47]. These indicator species analyses generate lists of target taxa that provide a more refined picture of seasonal changes in biodiversity—S9 Table lists all significant seasonally variable OTUs.

The years 2010 to 2014 showed changes in the Assemblage identified by several of the assays (Table 1); the pairwise analysis (S10 Table) identified when these changes occurred. The OTUs that most strongly characterise each year are presented in S11 Table. Six assays showed significant changes in Assemblage between 2010 and 2011 and each of the three subsequent years (S10 Table). In particular, the Assemblage from Copepod 1, Mollusca and Cnidaria assays responded strongly. This pattern suggests a biotic regime shift in response to an environmental anomaly. S11 Table lists all significant yearly variable OTUs.

### Biotic heatwave effects

The Rottnest Island area has global significance as it is situated within a site of high biodiversity that is largely endemic [36]. This sample set was particularly significant because it encompasses two uncharacteristic summer temperature extremes in 2011 and 2012. The WA marine heatwave was originally defined as occurring between November 2010 and April 2011 [38]. However, similarly high sea surface temperatures (SST) were recorded during the following year [48–50] (Fig 2B & S3 Fig). In this study, periods for the heatwaves were: “Heatwave 1”, a five-month heatwave, as described in Pearce and Feng (2013); and “Heatwave 2”, which encompasses Heatwave 1 and extends across a 17-month period from November 2010 –May 2012 (Fig 1B). The Assemblage from most assays (except Crustacea, Fish) responded significantly to the designated heatwave periods (Table 1).

The most significant changes in the Assemblage were between the periods pre- and post-Heatwave 1 (S12 Table). For Heatwave 2, significant differences were seen before, after, as well as during the thermal event (S12 Table). Analyses of both heatwave periods suggest that there were significant, and potentially persistent, changes that occurred within the zooplankton communities as a result of these collective temperature anomalies. Only ongoing research will determine whether these changes are permanent, however, climate-mediated change has already been reported in the same study area where Wernberg (et al.) [39] reported that a kelp dominated nearshore ecosystem shifted to a more tropicalised system containing seaweed turf.

The value of employing assays with different taxonomic specificities is shown by the lack of significant heatwave-induced Assemblage changes observed for some assays. No change was detected using the Crustacea and Fish assays. The taxa detected by these assays are generally long-lived with pelagic larval phases, so any significant change in these groups is likely to occur gradually and would only be detected with an even longer-term study. The Heatwaves had less significant effects on Richness, however the Copepod 1 and 3 assays demonstrated changes in Richness, particularly between before and after the thermal anomaly periods (S12 Table).

**Fig 3:**
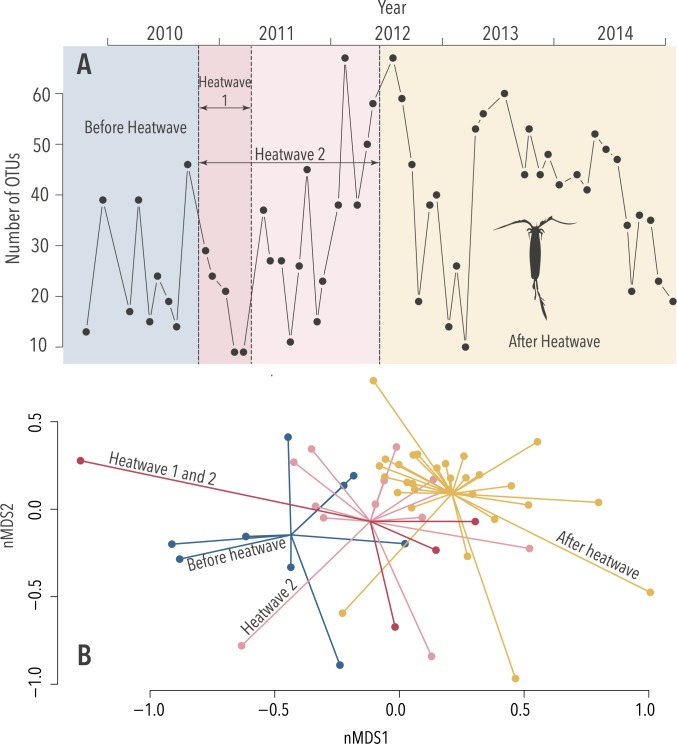
Heatwave effects revealed by Copepod 1 eDNA assay “Heatwave 1” and “Heatwave 2” are indicated. (A) Changes in the number of Operational Taxonomic Units (OTUs) over time (Richness: Heatwave 1; *p* < 0.01 & Heatwave 2; *p* < 0.05) and (B) changes in the diversity of the OTUs revealed by non-parametric multivariate analysis (Assemblage; *k* = 3, stress = 0.15, both *p* < 0.001), the coloured lines extrude from the centroids of each time period towards the variation in Assemblage from each sample.

The Copepod 1 assay illustrates the effects of Heatwaves 1 and 2 on the Assemblage and Richness (Fig 3). The Copepod 1 assay was designed *in silico* to focus on the genus *Triconia*, but, as is common in metabarcoding approaches, *in vitro*, the assay detects a much wider range of copepods as well as other arthropods.

OTUs characterising the periods defined by the heatwaves were identified by *indval* analysis. The OTUs corresponding to *Paracalanus indicus* (a copepod) and Pythiales (an order of water mould) are strong indicators for the ‘before’ periods (S13 & S14 Tables). The Copepod 1 OTUs characterising the heatwave ‘during’ periods were significantly different; only ten OTUs (11%) overlap. The best indicator for Heatwave 1 was Hexanauplia (the class which contains all copepods); this OTU is also an indicator for Heatwave 2 (S13 & S14 Tables). For the ‘after’ periods, nine OTUs are shared between them (15%). Nine anonymous copepod OTUs (15%) were strongly associated with the ‘after’ of both heatwave periods. This demonstrates the advantage of the OTU approach and provides an opportunity for taxonomists to link these sequences to the species that they provisionally represent.

These time-stamped metabarcoding data show, for the first time, that eDNA metabarcoding is able to track biotic shifts in response to seasonal and annual changes, as well as identify a known temperature anomaly that threatened global biodiversity hotspots on the west coast of Australia. This result has obvious implications for biomonitoring of oceans in the face of anthropogenic pressures including climate change, acidification, pollution, fishing and aquaculture impacts. The Assemblage and Richness data provided by eDNA metabarcoding can be integrated with other abiotic factors to develop a more holistic picture of how biomes respond to a variety of environmental factors.

### Biological response to abiotic change

Biological samples analysed in this study were collected alongside complementary measurements of physical and chemical characteristics of the sampling site. Sea surface temperature (SST) and the concentrations of salinity and silicate (an important nutrient in oceans), were all important explanatory abiotic variables for both Richness and Assemblage across the majority of metabarcoding assays (Table 2). These variables feature in either the ‘best’ or the most parsimonious alternative models for all of the assays used (Tables 2 and S15).

**Table 2:** Relationship between sea surface temperature (SST) and abiotic factors, and OTU richness (the number of OTUs in each sample)—nbGLM (negative binominal Generalised Linear Model; [52])—and assemblage (what OTUs are in each sample)—DistLM (Distance based Linear Model; [42])—as indicated by each assay.

Assay used	OTU diversity test	Variable	SST	Salinity	Silicate	Nitrate	Phosphate	Ammonium	Best Model	
Cnidaria	Assemblage	P	**<0.001**	**<0.001**	**<0.001**	0.072	0.114	0.001	R^2^	0.162
		R^2^	**0.053**	**0.067**	**0.072**	0.028	0.026	0.044		
	Richness	P	**0.069**	0.061	**0.029**	0.842	0.809	0.700	R^2^	0.112
		R^2^	**0.056 (+)**	0.059 (+)	**0.078 (-)**	< 0.001 (-)	0.001 (+)	0.002 (-)		
Copepod 1	Assemblage	P	**0.030**	**0.002**	**< 0.001**	0.097	0.020	0.100	R^2^	0.155
		R^2^	**0.034**	**0.050**	**0.079**	0.028	0.036	0.028		
	Richness	P	0.953	**0.045**	0.308	0.478	0.376	0.245	R^2^	0.067
		R^2^	< 0.001 (-)	**0.067 (-)**	0.018 (+)	0.009 (+)	0.014 (+)	0.024 (+)		
Copepod 2	Assemblage	P	**0.021**	**< 0.001**	**< 0.001**	0.141	0.264	0.008	R^2^	0.230
		R^2^	**0.011**	**0.126**	**0.120**	0.027	0.022	0.049		
	Richness	P	0.428	**< 0.001**	**< 0.001**	0.146	0.172	0.011	R^2^	0.309
		R^2^	0.004 (-)	**0.255 (-)**	**0.204 (+)**	0.036 (+)	0.032 (+)	0.102 (+)		
Copepod 3	Assemblage	P	**0.011**	**<0.001**	**<0.001**	0.043	0.447	0.002	R^2^	0.227
		R^2^	**0.042**	**0.138**	**0.092**	0.034	0.018	0.053		
	Richness	P	**0.537**	**<0.0001**	0.007	0.252	0.561	**0.045**	R^2^	0.392
		R^2^	**0.007 (+)**	**0.305 (-)**	0.115 (+)	0.023 (+)	0.006 (+)	**0.067 (+)**		
Crustacea	Assemblage	P	**0.001**	**<0.001**	0.005	0.337	0.479	0.009	R^2^	0.098
		R^2^	**0.046**	**0.056**	0.038	0.021	0.019	0.037		
	Richness	P	**0.079**	0.246	0.799	0.104	0.629	**0.015**	R^2^	0.183
		R^2^	**0.052 (+)**	0.083 (-)	0.001 (+)	0.045 (+)	0 .004 (-)	**0.096 (+)**		
Fish	Assemblage	P	**0.001**	**<0.001**	0.007	0.006	**0.313**	0.002	R^2^	0.147
		R^2^	**0.056**	**0.064**	0.043	0.044	**0.022**	0.049		
	Richness	P	0.976	**0.007**	**0.005**	**0.016**	**0.679**	0.035	R^2^	0.251
		R^2^	<0.001 (+)	**0.121 (+)**	**0.127 (-)**	**0.098 (-)**	**0.003 (+)**	0.077 (-)		
Mollusca	Assemblage	P	**<0.001**	**<0.001**	**<0.001**	0.023	0.068	**<0.001**	R^2^	0.197
		R^2^	**0.057**	**0.101**	**0.081**	0.033	0.028	**0.051**		
	Richness	P	**0.058**	0.750	0.391	0.730	0.248	0.465	R^2^	0.061
		R^2^	**0.061 (+)**	0.013 (+)	0.019 (-)	0.002 (+)	0.024 (-)	0.010 (+)		
Universal	Assemblage	P	**0.045**	**0.001**	**0.001**	0.026	0.010	0.014	R^2^	0.140
		R^2^	**0.034**	**0.061**	**0.059**	0.038	0.043	0.043		
	Richness	P	**0.299**	**0.045**	0.165	0.286	**0.044**	0.578	R^2^	0.212
		R^2^	**0.019 (+)**	**0.067 (-)**	0.034 (+)	0.020 (+)	**0.068 (+)**	0.006 (+)		

**Bolded type** indicates abiotic variables that belong to the most parsimonious model as selected using the AIC

+ or − indicate the direction of the relationship

SST and salinity explained a large portion of the biological variation we observed. The assay most sensitive to the abiotic factors was Copepod 3; where SST, and concentrations of salinity, and silicate explained 22.7% of the variation in Assemblage, and SST and salinity concentration explained 39.2% of the variation in Richness (Table 2).

Richness increased significantly with warmer SST for most assays, with the exception of Copepod 1 and Copepod 2, which showed an insignificant negative relationship to SST (Table 2). Richness conversely decreased with increasing salinity for the Copepod, Crustacean, and Universal assays, but reacted positively in the Cnidaria, Fish, and Mollusca results. Silicate correlations had the opposite pattern, being positively correlated with Richness in the Copepod, Crustacean, and Universal results, but negatively correlated to Richness when measured against the Cnidaria, Fish, and Mollusca assays (Table 2). These results are likely due to an indirect link between the environmental variables to the zooplankton composition via direct links upon the phytoplankton [51]. These results illustrate the different niches that zooplankton can exploit within an ecosystem. As one group of zooplankton find conditions uninhabitable and diminishes locally, another group will thrive within the niche.

### Conclusion

A recent editorial on marine monitoring [53] argued for a pressing need to make the shift from site-specific approaches to a functional, whole-sea system of monitoring. Here we show that eDNA metabarcoding is capable of responding to this challenge. Multi-year sample sets appropriate for eDNA analysis have not been previously available. Had this study been limited to a single point in time or even over the course of a year, where the longer-term patterns of change would be missed. Our study included two ‘marine heatwave’ periods and these data demonstrated that, using an effective eDNA metabarcoding toolkit, ecologically significant trends can be identified in response to a known environmental perturbation.

The biodiversity detected by our multi-assay eDNA metabarcoding ‘tool kit’ was vast, and while many barcodes could be assigned within the existing taxonomic framework, almost as many could not. While it could be argued that indicator species/OTUs should perhaps be the primary focus for taxonomic scrutiny employing both morphology and genetics, it is clear that as databases and assays improve, so too will the power of eDNA to identify the taxa present in complex ecosystems like this one. The results highlighted both the importance of collecting time-stamped samples (i.e. environmental biobanks [54]) and the significance of multi-gene metabarcoding for the long-term monitoring of marine ecosystems. For example, had only the universal 18S marker been used, much of the genetic depth of information would have been lost. While the 18S markers are typically longer and produce results across a broad range of taxa, it is more conserved than other barcodes and often results must be confined to a family level of identification. The study illustrates the need to balance the cost of the multi-marker approach with the amount of data that can be generated. The future implications of this data are that eDNA will generate much-needed baseline biotic data, and identify disturbance gradients, recovery profiles and potential ‘biotic tipping points’.

## Materials and methods

### Sampling

All sampling took place at the Rottnest Island National Reference Station (NRS), an Integrated Marine Observing System (IMOS [6]) site, Western Australia (Fig 1A). The site is situated at the midpoint of the sub-tropical zone of the Leeuwin current, approximately 20 km off the southwest coast of Western Australia. Abiotic sampling has occurred regularly at this site since 1951 and biological sampling by the IMOS program since 2008 [6]. The plankton sampling regime was instigated at this time and historically three separate monthly samples were taken; one for morphological analysis; one for biomass measurements and a third tow for later DNA analysis. We were provided access to these final samples.

Vertical plankton tows were taken on 55 occasions from October 2009 to January 2015, from the same site, in an almost regular monthly regime (Fig 1B). A 0.6 m wide, 3 m long drop net [55] with a100 μm mesh, which free falls at 1 ms^-1^, was dropped for 45 s. The seabed depth at the Rottnest Island sampling site is 50 m, so this sampling covered 90% of the water column. Plankton was collected on the downward fall; the motion of retrieval closes the net for the upward haul. The nets are washed in fresh water (with detergent if clogged), hung out to dry and stored dry between monthly sampling.

Samples were washed down and concentrated at the codend of the drop net and transferred into a sample jar using seawater. Samples were packed on ice until placed in long-term storage at -80°C immediately after return to the laboratory. Samples were later subsampled for this study and the sub-samples preserved at -20°C prior to DNA extraction.

### DNA extraction

Each plankton sample was homogenised, using a hand-held blender (OMNI Tip Homogenizer) and a hard tissue probe. About 20 μL of the resulting slurry was digested and extracted using DNAeasy Blood and Tissue kit (Qiagen) following the tissue protocol and a 2 x 100 μL elution in AE buffer. An extraction control was created during this phase. Extracts were stored at -20°C.

### Metabarcoding assay design

Over 20 group-specific PCR amplicon metabarcode assays were tested for use in this study. Sequences used for *in silico* assay design were downloaded from the National Center for Biotechnology Information (NCBI) GenBank database [56]. Database coverage was limited across all genes, so in most instances the cytochrome oxidase I (COI) gene provided the best option for metabarcoding.

Sequences were aligned in Geneious Version R8 and consensus sequences were derived from these alignments [57]. Sequences were examined for relatively conserved regions flanking 100-200bp hyper-variable targets (S4 Fig). This examination resulted in the creation of several new metabarcoding assays. These assays, along with some that were previously described, were then tested against 20 pilot plankton samples to determine which assays, when combined, produced the broadest coverage of taxa found within zooplankton (S16 Table). From these, eight assays, including five targeting COI (predominately, three for different copepods and one each for molluscs and cnidarians), one targeting 18S rRNA (“universal”) and two targeting16S rRNA (one each for actinopterygii and malacostraca), were selected for use in this study (S1 Table).

The 55 DNA extracts were assessed using qPCR for their response to each of the eight assays, which were applied to each sample’s neat extract and two dilutions (1/10 and 1/100). Extraction, non-template and positive controls (where available) were included for each assay. Each reaction comprised: 1 x Taq Gold buffer (Applied Biosystems [ABI], USA), 2 nM MgCl2 (ABI, USA), 0.4 mg/mL BSA (Fisher Biotec, Australia), 0.25 mM dNTPs (Astral Scientific, Australia), 0.4 μM each of forward and reverse primers (Integrated DNA Technologies, Australia), 0.6 μL of 1/10,000 SYBR Green dye (Life Technologies, USA), 1 U of *Taq* polymerase Gold (ABI, USA), 2 μL of DNA, and made up to 25 μL with PCR grade water. PCR conditions for all reactions included 95°C for 10 min followed by 50 cycles of 95°C for 30 sec, T_a_ (S1 Table) for 30 sec and 72°C for 45 sec, with a final extension of 72°C for 10 min. All reactions were set up in an ultra-clean laboratory used for trace and environmental DNA.

### Library builds & sequencing

Fusion tagged primers incorporating specific unique combinations of six to eight base pair MID (Multiplex IDentifier) tags, assay specific primers and Illumina adaptor sequences were assigned, in duplicate, to each DNA extract (and any negative control that produced a positive result during qPCR) in a single PCR step (giving a total of over 400 unique MID tagged combinations). Many samples are multiplexed within a single library and the MID tags allow for later separation and assignment of the individual sequences to their specific assays and samples. To prevent cross contamination within the NGS workflow, the MID tag primer combinations had not been used previously for marine samples and were not reused. Conditions for the fusion tagged PCR reactions were identical to the qPCR (above) and were carried out in duplicate, using the appropriate dilution determined by the qPCR. Reactions were monitored for efficient amplification by scrutinising qPCR dynamics. Tagged amplicons were combined in roughly equimolar concentrations to produce multiplexed sequencing libraries. On each library the fusion tags were not ‘saturated’, meaning that, while there are ten reverse tags to every forward tag, each run allowed for several unused forward and reverse combinations. If unused tag combinations are subsequently detected after sequencing, the tagging process is repeated to ensure there is no tag cross over. The libraries were then size-selected using a Pippin Prep (Sage Sciences, USA) instrument and quantified using a Lab Chip (PerkinElmer, USA). All sequencing was performed using Illumina’s MiSeq following the manufacturer’s protocol with the exception of the use of custom sequencing primers and with 20 pM PhiX, on either a Standard or Nano flow cell and 300–500 cycle kits.

### Taxonomic assignment

Sequences were assigned to the appropriate samples by their MID tags using Geneious R8 [57]. Initial filtering steps included ensuring the MID tags, gene specific primers and sequencing adapters, were all present in each sequence without error. Those sequences not matched were discarded from future analyses. The primers, adaptors and MID tags were removed from each of the sequences that passed these criteria, which were then filtered using a fastq filter (E_max > 0.5—USEARCH v8 [58]).

To increase the robustness of the data set, sequences were then separated into groups of unique sequences using USEARCH v8 [58]. Of these sequences, any group which contained < 1% of the total number of unique sequences was discarded—the filtered data are available for download on Data Dryad: doi:10.5061/dryad.sc673ds. This process, which may eliminate low abundance taxa, is conservative in that it ensures the removal of possible erroneous amplicons. Amplicons that passed the second filtering processes were queried against the National Center for Biotechnology Information (NCBI) GenBank nucleotide database [59] using BLASTn (Basic Local Alignment Search Tool [60]) with the default parameters and a reward of value of 1.

The search output files were imported into MEGAN v5 (METaGenome ANalyzer [61]) and visualised using the LCA (lowest common ancestor) parameters: min bitscore 100.0, and reports restricted to the best 5% of matches. Taxonomic assignment was considered only when the entire length of the query sequence matched the reference database. Taxonomic hierarchy was determined using the World Register of Marine Species [62]. Negative controls were all found to be clear with the exception of the 18S Universal assay, which showed some fungal contamination.

### Production of Operational Taxonomic Units (OTUs)

Clustering of similar sequences to produce OTUs was performed with USEARCH v8 [58]. The OTUs were formed from all filtered sequences from each assay using a 97% similarity threshold across all samples. The procedure also removed any potential chimeric sequences and any groups of unique sequences with an abundance of < 0.1% of the total number of unique sequences across all samples. Sequences discarded during this process were then mapped back on to existing OTUs to ensure the inclusion of all relevant data and those amplicons, which could not be mapped, were discarded. The OTUs were then assigned to the samples that they originated from and were converted to a presence/absence matrix. This approach also minimises any data misrepresentations as a result of potential unequal sequence amplification from marker choice or tag bias. The OTUs were statistically analysed in response to both temporal and abiotic factors.

### Statistical analysis

Statistical analyses, were performed using PERMANOVA+ [42] add on for Primer 7 [63] and R [64] with labdsv [45], and vegan [65]. The analyses were performed on the presence/absence OTU data matrix for the sequences obtained for each assay, thus allowing for all available genetic information to be taken into consideration. A total of 55 samples were used for analysis. The initial Pearson’s correlation test of the number of sequences produced by each assay, at each time point, and the number of OTUs was performed in R [64].

To prevent the inclusion of ‘outliers’ that might skew the results, the sequences for each assay were filtered to remove any OTUs that occurred only once in the study and also any samples that contained only one OTU. The richness and assemblage (genetic diversity) data for each sample were then examined using multivariate methods (PERMANOVA [66]) to test time-based relationships such as heatwave, seasonality and inter-annual effects). Annual and seasonal effects were tested using a nested design with three factors: Year (fixed, 5 levels), Season (nested in Year, random), and Month (Nested in Season, random). Tests for heatwave effects were conducted using a single factor (fixed, either 5 month or 17 month heatwave window) with three levels (before, during, after). To illustrate these patterns, two-dimensional nonmetric multidimensional scaling (nMDS) plots were formed in R (package vegan).

The indicator species that were characteristic of years, seasons, and heatwave events were identified using indval analyses in R (package labdsv). The indval indicator value is calculated using a combination of the fidelity of an OTU to a time period and the frequency at which it occurs during that same time period. All pairwise comparisons were performed using PERMANOVA.

The role of abiotic variables in explaining variation in both the multivariate OTU assemblage, and the univariate OTU richness was analysed with linear models for each assay. Multivariate analysis was done using distance based linear models (DistLM) in PERMANOVA+. Bray-Curtis similarity matrices were constructed from the presence/absence OTU data. The abiotic variables sea surface temperature (SST) and concentrations of salinity, silicate, nitrate, phosphate, and ammonium were available for selection by the model. The ‘best’ selection procedure and the AIC selection criteria were used to select the model that best explained the variation in the OTU assemblage that was recorded for each assay. The best alternative models for each number of variables that were within 2 AIC of the selected model were also reported (S15 Table).

Univariate OTU richness was analysed for each assay with generalised linear models (GLMs) fitted in R using the functions glm [64] and glm.nb [52]. The abiotic explanatory variables available were the same as those above. During analysis the distribution of the residuals of each model were plotted and examined to select the appropriate distribution. In all cases the negative binomial distribution with a log link was used [67]. The model with the lowest AIC was selected using the best of both forward and backward selection procedures. Models within 2AIC of the selected model were also reported. To aid in the interpretation of the relationship between each abiotic variable and the OTU assemblage composition and richness were also calculated and reported for each abiotic variable.

## Supporting information

S1 Table The metabarcoding PCR assays used in this study. (PDF)

S2 Table Number of Arthropoda detections in Rottnest Island zooplankton samples by each assay. (PDF)

S3 Table Number of Chordata detections in Rottnest Island zooplankton samples by each assay. (PDF)

S4 Table Number of Mollusca detections in Rottnest Island zooplankton samples by each assay. (PDF)

S5 Table Number of Cnidaria detections in Rottnest Island zooplankton samples by each assay. (PDF)

S6 Table Number of Echinodermata detections in Rottnest Island zooplankton samples by each assay. (PDF)

S7 Table Number of other Animalia taxa detections in Rottnest Island zooplankton samples by each assay. (PDF)

S8 Table Pairwise analysis of seasonal OTU richness & assemblage, t statistics included for significant results (t)—PERMANOVA+[7]. (PDF)

S9 Table Indicator species analysis for seasonal variation—*Indval* [8]. (PDF)

S10 Table Pairwise analysis of yearly OTU richness & assemblage, t statistics included for significant results (t)—PERMANOVA+[7]. (PDF)

S11 Table Indicator species analysis for yearly variation—*Indval* [8]. (PDF)

S12 Table Pairwise analysis of the OTU richness & assemblage between before, during and after the heatwaves, t statistics included for significant results (t)—PERMANOVA+[7]. (PDF)

S13 Table Indicator species analysis for five-month heatwave variation—*Indval* [8]. (PDF)

S14 Table Indicator species analysis for 17-month heatwave variation—*Indval* [8]. (PDF)

S15 Table Alternative linear models for Assemblage and Richness. (PDF)

S16 Table Metabarcoding PCR assays developed and tested for this study. (PDF)

S1 Fig Example of typical rarefaction curves. These were produced, using all eight assays, from a single sample taken on May 23 2012
(EPS)

S2 Fig Number of sequences (beige—right axis) and number of OTUs (red—left axis), per sample, for each assay. The assays showed a range of correlations between sequencing depth and the number of OTUs produced. No correlation was detected in the 18S and Crustacea 16S assays, while the Fish 16S showed a weak but non-significant correlation. The COI assays all produced moderate correlations.
(EPS)

S3 Fig Annual sea surface temperature anomalies from 1900–2017. The 2011 and 2012 heatwave events produce the two highest peaks—Extracted from the Bureau of Meteorology time series graphs [9].
(PDF)

S4 Fig Example of a consensus alignment used to create the assays used in this study. This COI alignment resulted in the Copepod 3 assay [10].
(EPS)

S1 Text References used for supporting information. (PDF)
