# Supplementary material for: Marine environmental DNA biomonitoring reveals seasonal patterns in biodiversity and identifies ecosystem responses to anomalous climatic events
Source: PLoS Genet. 2019 Feb 8;15(2):e1007943. doi: 10.1371/journal.pgen.1007943 (PMC6368286; doi:10.1371/journal.pgen.1007943)
Supplement: S1 Table — (PDF) [file pgen.1007943.s001.pdf]

Table S1: The metabarcoding PCR assays used in this study

PCR assay	Primer set used	Primary target taxa	Gene	Primer sequence	Amplicon length (bp)	Reference	Assay Ta (°C)
Cnidaria	Cnidaria_F<br>Cnidaria_R	Cnidarians	COI	5′ CATGATHTTYTTCWTDGTMATGCC 3′<br>5′ GTYCAWCCWGTWCCWRCYCC 3′	~145	This study	52
Copepod 1	Triconia_F<br>Triconia_R	Copepods	COI	5′ CAGGVTCTTTAYTWGGRGATG 3′<br>5′ AAAATCTTATATTATTTARBCGRGG 3′	~131	This study	49
Copepod 2	Lucicutia_F<br>Lucicutia_R	Copepods	COI	5′ CCHGAYATAGCTTTYCCHCG 3′<br>5′ GAAAAAAATTGCAAAATCTACDGATC 3′	~134	This study	52
Copepod 3	Acartia_F<br>Acartia_R	Copepods	COI	5′ GGRGAYGATCARRTYTATAAYGT 3′<br>5′ TTYATWCGWGGAAAHGCYATRTC 3′	~103	This study	50
Crustacea	Crust16S_ F (short)<br>Crust16S_ R (short)	Crustaceans	16S rRNA	5′ GGGACGATAAGACCCTATA 3′<br>5′ ATTACGCTGTTATCCCTAAAG 3′	~170	Berry et al. [1]	51
Mollusca	Limacina_F<br>Limacina_R	Molluscs	COI	5′ TAATTGGNGGVTTTGGRAAYTG 3′<br>5′ GTTCAHCCTRAYCCTRCNCC 3′	~118	This study	52
Fish	16s2R (degenerate)<br>Fish16sF/D	Actinopterygii	16S rRNA	5′CGCTGTTATCCCTADRGTAACT 3′<br>5′ GACCCTATGGAGCTTTAGAC 3′	~200	F- Deagle et al. [2]<br>R- Berry et al. [1]	54
Universal	18s_1F<br>18s_400R	Universal	18S rRNA	5′ GCCAGTAGTCATATGCTTGTCT 3′<br>5′ GCCTGCTGCCTTCCTT 3′	~400	Pochon et al. [3]	52

Table 1: Primer sets used in this study: 'F' refers to the forward primer; 'R' refers to the reverse primer.
