# Supplementary material for: Marine environmental DNA biomonitoring reveals seasonal patterns in biodiversity and identifies ecosystem responses to anomalous climatic events
Source: PLoS Genet. 2019 Feb 8;15(2):e1007943. doi: 10.1371/journal.pgen.1007943 (PMC6368286; doi:10.1371/journal.pgen.1007943)
Supplement: S2 Table — (PDF) [file pgen.1007943.s002.pdf]

Table S2: Number of Arthropoda detections in Rottnest Island zooplankton samples by each assay.

Class	Order	Family	Genus	Species	Australia [4, 5]	Rottnest [5]	Copepod 3	Cnidaria	Crust	Mollusca	Copepod 2	Copepod 1	18S
Branchiopoda - Diplostraca					Yes	Yes	1	0	0	0	0	0	0
Branchiopoda		Limnadiidae (ALA)			Yes	Yes	1	0	0	0	0	0	0
	Ctenopoda	Sididae	*Penilia*	*Penilia avirostris*	Yes	Yes	32	24	0	31	0	0	14
	Onychopoda	Podonidae	*Evadne*	*Evadne spinifera*	Yes	Yes	0	0	1	0	0	0	0
			Pseudevadne		Yes	Yes	0	0	0	4	0	0	0
				Pseudevadne tergestina	Yes	Yes	0	6	0	0	0	0	0
Hexanauplia	Calanoida				Yes	Yes	115	15	0	77	222	2	125
		Acartiidae			Yes	Yes	0	0	0	0	0	10	0
			Acartia		Yes	Yes	0	0	0	0	0	0	10
			Acartia (Acartia)	*Acartia (Acartia) danae*	Yes	Yes	20	0	0	1	0	0	1
				Acartia (Acartia) negligens	Yes	Yes	46	17	0	1	0	0	0
			Acartiella		No	No	0	0	0	0	32	0	0
		Aetideidae			Yes	Yes	0	0	0	0	6	0	25
			Aetideus	*Aetideus acutus*	Yes	No	0	0	0	0	4	0	0
			Gaetanus		Yes	No	0	0	0	0	0	0	17
		Augaptilidae	*Haloptilus*		Yes	Yes	0	0	0	0	0	0	4
				Haloptilus longicornis	Yes	Yes	0	0	0	0	2	0	0
		Calanidae			Yes	Yes	2	1	0	0	0	8	17
			Canthocalanus	*Canthocalanus pauper*	Yes	Yes	14	0	0	17	0	0	0
			Cosmocalanus		Yes	Yes	2	0	0	0	0	1	0
				Cosmocalanus darwinii	Yes	Yes	36	1	0	3	0	42	0
			Nannocalanus		Yes	Yes	1	0	0	5	0	3	0
			Neocalanus	*Neocalanus gracilis*	Yes	Yes	0	0	0	1	0	0	0
			Undinula	*Undinula vulgaris*	Yes	Yes	17	0	0	1	0	2	7
		Candaciidae			Yes	Yes	4	0	0	0	0	0	0
			Candacia		Yes	Yes	0	0	0	0	0	0	10
				Candacia bipinnata	Yes	Yes	0	0	0	2	0	0	0
				Candacia bradyi	Yes	Yes	0	0	0	40	2	0	0

Class	Order	Family	Genus	Species	Australia [4, 5]	Rottnest [5]	Copepod 3	Cnidaria	Crust	Mollusca	Copepod 2	Copepod 1	18S
				Candacia catula	Yes	Yes	0	0	0	2	1	0	0
				Candacia discaudata	Yes	No	0	0	0	2	1	0	0
				Candacia pachydactyla	Yes	No	0	0	0	0	0	0	1
				Candacia simplex	Yes	No	1	0	0	0	0	0	0
				Candacia truncata	Yes	No	5	0	0	0	0	0	0
		Centropagidae			Yes	Yes	0	0	0	0	0	2	0
			Boeckella		Yes	Yes	0	0	0	2	0	0	0
			Centropages	*Centropages furcatus*	Yes	No	9	0	0	2	0	0	0
				Centropages orsinii	Yes	Yes	7	0	0	1	2	0	0
		Clausocalanidae	*Clausocalanus*		Yes	Yes	5	0	0	0	0	14	44
				Clausocalanus arcuicornis	Yes	Yes	6	0	0	0	0	0	0
				Clausocalanus farrani	Yes	Yes	0	0	0	1	0	34	0
				Clausocalanus furcatus	Yes	Yes	54	3	0	21	53	0	0
				Clausocalanus ingens	Yes	No	1	0	0	0	0	0	4
				Clausocalanus jobei	Yes	Yes	2	0	0	0	2	23	0
				Clausocalanus lividus	Yes	No	0	0	0	0	3	0	0
				Clausocalanus mastigophorus	Yes	Yes	0	0	0	0	1	0	0
				Clausocalanus minor	Yes	Yes	6	0	0	0	25	15	0
				Clausocalanus parapergens	Yes	No	1	0	0	0	23	4	0
				Clausocalanus pergens	Yes	No	1	0	0	0	0	0	0
			Ctenocalanus	*Ctenocalanus vanus*	Yes	Yes	16	0	0	0	7	32	0
		Eucalanidae			Yes	Yes	9	0	0	3	0	0	20
			Eucalanus		Yes	No	0	0	0	0	1	0	0
			Pareucalanus	*Pareucalanus attenuatus*	No	No	0	3	0	0	0	0	0
				Pareucalanus sewelli	Yes	No	3	0	0	0	0	0	0
		Euchaetidae			Yes	Yes	0	0	0	0	0	1	0
			Euchaeta	*Euchaeta concinna*	Yes	No	4	0	0	0	0	2	0
				Euchaeta longicornis	No	No	0	0	0	1	0	0	0

Class	Order	Family	Genus	Species	Australia [4, 5]	Rottnest [5]	Copepod 3	Cnidaria	Crust	Mollusca	Copepod 2	Copepod 1	18S
				Euchaeta marina	Yes	No	0	0	0	9	11	0	0
		Lucicutiidae	*Lucicutia*	*Lucicutia flavicornis*	Yes	Yes	0	0	0	31	29	0	0
		Metridinidae	*Pleuromamma*	*Pleuromamma gracilis*	Yes	Yes	0	0	0	0	7	0	0
				Pleuromamma piseki	Yes	Yes	0	0	0	0	9	0	0
		Paracalanidae			Yes	Yes	0	0	0	0	16	8	1
			Acrocalanus		Yes	Yes	0	0	0	0	2	0	0
				Acrocalanus gracilis	Yes	Yes	11	0	0	0	2	1	0
				Acrocalanus monachus	Yes	No	0	0	0	0	0	0	10
			Bestiolina		Yes	Yes	38	0	0	29	0	0	0
			Calocalanus		Yes	Yes	25	0	0	1	48	1	0
				Calocalanus minutus	No	No	19	0	0	0	0	0	0
				Calocalanus pavo	Yes	Yes	11	0	0	14	3	8	0
				Calocalanus plumulosus	Yes	Yes	13	0	0	0	28	0	14
			Delibus		Yes	No	22	0	0	34	29	13	0
			Mecynocera	*Mecynocera clausi*	Yes	Yes	0	0	0	40	0	0	4
			Paracalanus		Yes	Yes	43	0	0	10	58	4	0
				Paracalanus aculeatus	Yes	Yes	45	0	0	0	0	0	0
				Paracalanus denudatus	Yes	No	0	0	0	0	21	29	0
				Paracalanus gracilis	No	No	0	0	0	0	17	0	0
				Paracalanus indicus	Yes	Yes	55	0	0	55	0	8	0
				Paracalanus nanus	No	No	41	0	0	4	30	5	0
				Paracalanus parvus	Yes	Yes	0	0	0	0	0	0	54
		Pontellidae	*Labidocera*	*Labidocera acuta*	Yes	No	0	2	0	1	0	0	0
				Labidocera minuta	Yes	Yes	33	0	0	2	0	0	0
			Pontellina	*Pontellina plumata*	Yes	No	1	11	0	1	0	0	0
		Rhincalanidae	*Rhincalanus*		Yes	Yes	1	0	0	0	0	0	0
				Rhincalanus cornutus	Yes	No	0	0	0	1	0	0	0
		Scolecitrichidae	*Scolecithricella*		Yes	No	1	0	0	0	0	0	0
				Scolecithricella longispinosa	No	No	1	0	0	0	2	0	0
			Scolecithrix	*Scolecithrix danae*	Yes	Yes	17	0	0	0	0	0	0

Class	Order	Family	Genus	Species	Australia [4, 5]	Rottnest [5]	Copepod 3	Cnidaria	Crust	Mollusca	Copepod 2	Copepod 1	18S
		Subeucalanidae	*Subeucalanus*		Yes	Yes	5	0	0	10	0	0	4
				Subeucalanus mucronatus	Yes	No	2	1	0	3	1	0	0
				Subeucalanus pileatus	Yes	Yes	1	0	0	9	0	0	0
				Subeucalanus subtenuis	Yes	No	42	0	0	0	0	0	0
		Temoridae	*Temora*		Yes	Yes	0	0	0	0	0	0	5
				Temora discaudata	Yes	Yes	1	0	0	5	1	0	0
	Cyclopoida - Poecilostomatoida				Yes	Yes	0	0	0	3	2	2	0
	Cyclopoida	Corycaeidae	*Farranula*	*Farranula gibbula*	Yes	Yes	0	0	0	7	0	0	0
		Oncaeidae	*Oncaea*		Yes	Yes	0	0	0	2	0	8	0
				Oncaea mediterranea	Yes	Yes	0	0	0	6	0	10	0
				Oncaea scottodicarloi	Yes	No	0	0	0	3	0	12	0
				Oncaea waldemari	Yes	No	0	1	0	4	0	36	0
			Triconia		Yes	Yes	0	0	0	4	0	27	0
				Triconia minuta	No	No	0	0	0	0	0	9	0
				Triconia umerus	Yes	No	0	0	0	0	0	17	0
		Sapphirinidae			Yes	Yes	0	0	0	2	1	0	0
			Copila	*Copilia mirabilis*	Yes	Yes	0	0	0	12	0	0	0
			Sapphirina	*Sapphirina metallina*	Yes	No	0	0	0	1	1	0	0
	Harpacticoida	Euterpinidae	*Euterpina*	*Euterpina acutifrons*	Yes	Yes	0	0	0	0	0	0	1
	Lepadiformes	Lepadidae	*Conchoderma*	*Conchoderma virgatum*	Yes	No	2	2	0	1	0	0	0
Insecta	Coleoptera	Hydrophilidae			Yes	Yes	0	1	0	0	0	0	0
Malacostraca	Amphipoda				Yes	Yes	0	0	0	1	0	0	0
		Ischyroceridae	*Jassa*	*Jassa slatteryi*	Yes	No	1	0	0	0	0	0	0
		Lestrigonidae	*Hyperietta*		Yes	Yes	2	0	0	0	0	0	0
				Hyperietta parviceps	No	No	0	0	0	2	0	0	0
			Hyperioides	*Hyperioides longipes*	Yes	No	1	0	0	0	0	0	0
				Hyperioides sibaginis	Yes	No	2	0	0	1	0	0	0
			Lestrigonus	*Lestrigonus bengalensis*	Yes	Yes	0	0	0	0	0	2	0
	Decapoda - Anomura				Yes	Yes	2	0	0	0	0	0	0

Class	Order	Family	Genus	Species	Australia [4, 5]	Rottnest [5]	Copepod 3	Cnidaria	Crust	Mollusca	Copepod 2	Copepod 1	18S
	Decapoda - Caridea				Yes	Yes	1	0	0	0	0	0	0
	Decapoda - Pilumnoidea				Yes	Yes	0	0	41	0	0	0	0
	Decapoda - Xanthoidea				Yes	Yes	1	0	0	0	0	0	0
	Decapoda				Yes	Yes	2	5	6	5	0	0	20
		Alpheidae			Yes	Yes	0	0	0	19	0	0	0
			Alpheus		Yes	Yes	0	0	0	0	0	0	2
		Axiidae	*Spongiaxius*	*Spongiaxius brucei*	Yes	No	0	0	0	0	0	0	1
		Callianassidae			Yes	Yes	0	0	0	0	0	0	1
			Trypaea	*Trypaea australiensis*	Yes	No	0	0	3	0	0	0	0
		Diogenidae	*Calcinus*	*Calcinus dapsiles*	Yes	Yes	10	0	9	0	0	0	0
		Epialtidae	*Menaethius*		Yes	No	0	0	4	0	0	0	0
		Hippolytidae			Yes	Yes	0	0	1	0	0	0	1
			Alope	*Alope orientalis*	Yes	Yes	0	0	5	0	0	0	0
			Hippolyte	*Hippolyte bifidirostris*	No	No	0	0	0	0	0	0	2
		Hymenosomatidae	*Halicarcinus*		Yes	Yes	0	0	1	1	0	0	1
		Luciferidae	*Belzebub*	*Belzebub intermedius*	No	No	5	0	0	0	0	0	0
			Lucifer		Yes	Yes	0	0	0	10	0	0	0
				Lucifer typus	Yes	No	3	0	0	0	0	0	0
		Lysmatidae	*Lysmata*	*Lysmata ternatensis*	No	No	0	0	1	0	0	0	0
		Palinuridae			Yes	Yes	1	0	0	0	0	0	0
			Panulirus	*Panulirus cygnus*	Yes	Yes	0	0	4	0	0	0	0
		Pandalidae			Yes	Yes	0	0	0	0	0	0	1
		Panopeidae			Yes	No	1	0	0	0	0	0	0
			Eurytium		No	No	2	0	0	0	0	0	0
		Pasiphaeidae	*Leptochela*		Yes	Yes	0	0	0	0	0	0	3
		Penaeidae			Yes	Yes	0	0	2	0	0	0	0
		Polybiidae			Yes	Yes	0	0	3	0	0	0	0
			Liocarcinus	*Liocarcinus corrugatus*	Yes	Yes	2	0	0	0	0	0	0
		Portunidae	*Thalamita*		Yes	Yes	0	0	4	0	0	0	0
				Thalamita admete	Yes	No	0	0	3	0	0	0	0
		Processidae	*Nikoides*		Yes	No	0	0	2	0	0	0	0

Class	Order	Family	Genus	Species	Australia [4, 5]	Rottnest [5]	Copepod 3	Cnidaria	Crust	Mollusca	Copepod 2	Copepod 1	18S
				Nikoides danae	Yes	No	0	0	0	0	0	0	4
		Pseudoziidae			Yes	No	0	0	0	1	0	0	0
		Scyllaridae	*Crenarctus*	*Crenarctus bicuspidatus*	No	No	0	0	1	0	0	0	0
		Xanthidae			Yes	Yes	0	0	3	0	0	0	0
			Actaeodes		Yes	Yes	0	0	7	0	0	0	0
			Danielea	*Danielea noelensis*	No	No	0	0	1	0	0	0	0
			Nanocassiope		Yes	Yes	3	0	0	0	0	0	0
				Nanocassiope alcocki	Yes	No	1	0	11	0	0	0	0
			Paraxanthias	*Paraxanthias elegans*	Yes	No	0	0	2	0	0	0	0
	Euphausiacea				Yes	Yes	0	0	6	0	0	0	0
		Euphausiidae			Yes	Yes	0	0	0	0	0	0	2
			Euphausia		Yes	Yes	0	0	0	1	0	0	0
				Euphausia hemigibba	Yes	No	4	0	0	3	0	0	0
				Euphausia recurva	Yes	Yes	4	0	7	0	0	0	0
			Stylocheiron	*Stylocheiron carinatum*	Yes	No	0	1	0	0	0	0	0
	Sessilia				Yes	Yes	0	1	0	0	0	0	0
Ostracoda	Halocyprida	Halocyprididae			Yes	Yes	0	0	0	1	0	0	0
	Myodocopida	Cypridinidae			Yes	Yes	0	0	0	0	0	0	5
