# Supplementary material for: Marine environmental DNA biomonitoring reveals seasonal patterns in biodiversity and identifies ecosystem responses to anomalous climatic events
Source: PLoS Genet. 2019 Feb 8;15(2):e1007943. doi: 10.1371/journal.pgen.1007943 (PMC6368286; doi:10.1371/journal.pgen.1007943)
Supplement: S3 Table — (PDF) [file pgen.1007943.s003.pdf]

Table S3: Number of Chordata detections in Rottnest Island zooplankton samples by each assay.

Class	Order	Family	Genus	Species	In Australia [5]	Rottnest [5]	Copepod 3	Cnidaria	Fish	Mollusca	Copepod 1	18S
Actinopterygii	Anguilliformes	Congridae	*Gnathophis*		Yes	Yes	1	0	0	0	0	0
		Muraenidae			Yes	Yes	0	0	1	0	0	0
	Aulopiformes	Aulopidae			Yes	Yes	2	0	0	0	0	0
			Latropiscis	*Latropiscis purpurissatus*	Yes	Yes	0	0	1	0	0	0
		Notosudidae	*Scopelosaurus*		Yes	Yes	1	0	0	0	0	0
	Beryciformes	Berycidae	*Centroberyx*		Yes	Yes	0	0	1	0	0	0
				Centroberyx lineatus	Yes	Yes	1	0	0	1	0	0
	Clupiformes	Clupeidae	*Sardinops*	*Sardinops sagax*	Yes	Yes	31	30	37	31	0	0
			Spratelloides		Yes	Yes	0	0	1	0	0	0
		Dussumieriidae	*Etrumeus*		Yes	Yes	0	0	0	2	0	0
				Etrumeus teres	Yes	Yes	10	12	20	11	0	0
		Engraulidae	*Engraulis*		Yes	Yes	0	0	16	2	0	0
				Engraulis japonicus	No	No	8	0	0	0	0	0
	Gadiformes	Moridae			Yes	Yes	0	0	2	0	0	0
			Lotella	*Lotella rhacina*	Yes	Yes	2	0	1	0	0	0
	Myctophiformes				Yes	Yes	0	0	1	0	0	0
		Myctophidae			Yes	Yes	0	0	1	0	0	0
			Benthosema	*Benthosema fibulatum*	Yes	Yes	0	0	1	0	0	0
			Diaphus		Yes	Yes	1	0	6	0	0	0
			Lampanyctus	*Lampanyctus alatus*	Yes	Yes	1	0	0	0	0	0
			Notolychnus	*Notolychnus valdiviae*	Yes	Yes	0	0	1	0	0	0
			Scopelopsis	*Scopelopsis multipunctatus*	Yes	Yes	1	0	0	0	0	0
	Perciformes	Apogoninae			Yes	Yes	1	0	0	0	0	0
			Apogon	*Apogon doederleini*	Yes	Yes	1	1	0	1	0	0
		Arripidae	*Arripis*	*Arripis trutta*	Yes	Yes	0	0	1	0	0	0
		Blennidae	*Cirripectes*	*Cirripectes castaneus*	Yes	No	1	0	0	0	0	0
		Carangidae	*Pseudocaranx*	*Pseudocaranx wrighti*	Yes	Yes	1	0	2	0	0	0
			Trachurus		Yes	Yes	0	0	8	1	0	0
		Cheilodactylidae	*Nemadactylus*		Yes	Yes	0	0	2	0	0	0
		Emmelichthyidae	*Plagiogeneion*		Yes	Yes	1	0	0	0	0	0
				Plagiogeneion rubiginosum	Yes	Yes	0	0	1	0	0	0

Class	Order	Family	Genus	Species	In Australia [5]	Rottnest [5]	Copepod 3	Cnidaria	Fish	Mollusca	Copepod 1	18S
		Gerreidae	*Parequula*		Yes	Yes	0	0	1	0	0	0
				Parequula melbournensis	Yes	Yes	0	0	3	0	0	0
		Glaucosomatidae	*Glaucosoma*	*Glaucosoma hebraicum*	Yes	Yes	0	0	1	0	0	0
		Gobiidae			Yes	Yes	0	0	1	0	0	0
		Haemulidae			Yes	Yes	0	0	1	0	0	0
		Kyphosidae	*Kyphosus*		Yes	Yes	0	0	1	0	0	0
		Labridae			Yes	Yes	5	0	4	0	0	0
			Austrolabrus	*Austrolabrus maculatus*	Yes	Yes	0	0	16	0	0	0
			Choerodon		Yes	Yes	0	0	1	0	0	0
			Coris		Yes	Yes	0	0	2	0	0	0
				Coris auricularis	Yes	Yes	1	0	0	0	0	0
			Halichoeres	*Halichoeres brownfieldi*	Yes	Yes	0	0	4	0	0	0
			Labroides	*Labroides dimidiatus*	Yes	Yes	0	0	1	0	0	0
			Notolabrus		Yes	Yes	1	0	0	0	0	0
			Ophthalmolepis	*Ophthalmolepis lineolata*	Yes	Yes	0	0	1	0	0	0
			Pictilabrus		Yes	Yes	0	0	15	0	0	0
			Pseudolabrus	*Pseudolabrus biserialis*	Yes	Yes	0	0	1	0	0	0
			Thalassoma		Yes	Yes	0	0	1	0	0	0
		Mullidae			Yes	Yes	0	0	0	11	0	0
			Upeneichthys		Yes	Yes	0	0	2	0	0	0
				Upeneichthys stotti	Yes	Yes	0	0	10	0	0	0
				Upeneichthys vlamingii	Yes	Yes	0	0	0	1	0	0
		Odacidae			Yes	Yes	0	0	3	0	0	0
			Heteroscarus	*Heteroscarus acroptilus*	Yes	Yes	3	0	0	0	0	0
			Odax	*Odax acroptilus*	Yes	Yes	0	0	3	0	0	0
		Opistognathidae			Yes	Yes	0	0	1	0	0	0
		Pempheridae	*Parapriacanthus*		Yes	Yes	0	0	3	0	0	0
				Parapriacanthus elongatus	Yes	Yes	0	0	11	0	0	0
		Pinguipedidae	*Parapercis*	*Parapercis ramsayi*	Yes	Yes	0	0	1	0	0	0
		Pomacentridae	*Chromis*		Yes	Yes	4	0	5	0	0	0
				Chromis notata	No	No	4	0	4	0	0	0
		Scaridae	*Scarus*	*Scarus chameleon*	Yes	Yes	1	0	0	0	0	0

Class	Order	Family	Genus	Species	In Australia [5]	Rottnest [5]	Copepod 3	Cnidaria	Fish	Mollusca	Copepod 1	18S
		Scombridae	*Scomber*		Yes	Yes	1	0	1	0	0	0
		Sillaginidae	*Sillaginodes*	*Sillaginodes punctatus*	Yes	Yes	0	0	2	0	0	0
			Sillago		Yes	Yes	1	0	1	0	0	0
				Sillago bassensis	Yes	Yes	1	0	1	0	0	0
		Sparidae			Yes	Yes	0	0	1	0	0	0
	Scorpaeniformes				Yes	Yes	0	0	1	0	0	0
		Neosebastidae	*Neosebastes*		Yes	Yes	0	0	2	0	0	0
		Platycephalidae	*Leviprora*	*Leviprora inops*	Yes	Yes	0	0	0	1	0	0
			Platycephalus		Yes	Yes	0	0	5	0	0	0
			Thysanophrys	*Thysanophrys cirronasa*	Yes	Yes	0	0	0	1	0	0
		Triglidae	*Lepidotrigla*		Yes	Yes	0	0	2	0	0	0
	Stomiiformes	Gonostomatidae	*Cyclothone*		Yes	Yes	0	0	1	0	0	0
		Phosichthyidae	*Vinciguerria*		Yes	Yes	3	0	0	1	0	0
	Tetraodontiformes	Monacanthidae			Yes	Yes	3	0	3	0	0	0
			Acanthaluteres		Yes	Yes	0	1	0	0	0	0
				Acanthaluteres vittiger	Yes	Yes	0	0	4	0	0	0
			Eubalichthys	*Eubalichthys mosaicus*	Yes	Yes	2	1	1	0	1	0
			Nelusetta	*Nelusetta ayraudi*	Yes	Yes	1	0	1	0	0	0
			Scobinichthys	*Scobinichthys granulatus*	Yes	Yes	0	0	1	0	0	0
Amphibia	Anura	Ranidae			Yes	No	1	0	0	0	0	0
Appendicularia	Copelata	Oikopleuridae	*Oikopleura*		Yes	No	0	0	0	0	0	1
				Oikopleura dioica	Yes (6)	No	0	0	0	0	0	10
Ascidiacea	Phlebobrancia	Perophoridae			Yes	Yes	0	0	0	0	0	1
	Stolidobranchia	Pyuridae	*Herdmania*	*Herdmania momus*	Yes	Yes	0	0	0	0	0	1
Thaliacea	Salpida	Salpidae			Yes	Yes	0	0	0	0	0	1
			Brooksia	*Brooksia rostrata*	Yes	No	0	0	0	0	0	1
			Cyclosalpa		Yes	No	0	0	0	0	0	1
			Thalia		Yes	No	0	0	0	0	0	8
	Doliolida	Doliolidae			Yes	Yes	0	0	0	0	0	1
			Doliolum	*Doliolum nationalis*	Yes (6)	No	1	0	0	0	0	0
