# Supplementary material for: Marine environmental DNA biomonitoring reveals seasonal patterns in biodiversity and identifies ecosystem responses to anomalous climatic events
Source: PLoS Genet. 2019 Feb 8;15(2):e1007943. doi: 10.1371/journal.pgen.1007943 (PMC6368286; doi:10.1371/journal.pgen.1007943)
Supplement: S4 Table — (PDF) [file pgen.1007943.s004.pdf]

Table S4: Number of Mollusca detections in Rottnest Island zooplankton samples by each assay.

Class	Order	Family	Genus	Species	In Australia [5]	Rottnest [5]	Copepod 3	Cnidaria	Mollusca	Copepod 2	Copepod 1	18S
Bivalvia	Ostreida	Ostreidae			Yes	Yes	0	1	0	0	0	0
		Pinnidae			Yes	Yes	0	0	1	0	0	0
	Pterioida (ALA)				Yes	No	0	0	2	0	0	0
Cephalopoda	Decapodiformes	Idiosepiidae			Yes	Yes	0	0	1	0	0	0
Gastropoda - Hypsogastropoda					Yes	Yes	0	0	0	0	14	0
Gastropoda					Yes	Yes	0	0	0	0	6	4
		Acteonidae	*Pupa*		Yes	Yes	0	0	1	0	0	0
		Aplustridae	*Micromelo*	*Micromelo undatus*	Yes	No	0	1	0	0	1	0
		Lepetidae			Yes	No	1	0	0	0	0	0
		Lottiidae			Yes	Yes	1	0	0	0	0	0
		Pyramidellidae	*Cingulina*		Yes	Yes	0	0	1	0	0	0
	Anaspidea	Aplysiidae			Yes	Yes	0	0	3	0	0	0
			Aplysia	*Aplysia gigantea*	Yes	Yes	0	0	2	0	0	0
			Dolabella	*Dolabella auricularia*	Yes	Yes	0	1	0	0	0	0
	Cephalaspidea	Bullidae	*Bulla*	*Bulla quoyii*	Yes	Yes	0	0	4	0	0	0
	Caenogastropoda				Yes	Yes	0	0	0	0	5	2
		Cerithiidae	*Cacozeliana*	*Cacozeliana granarium*	Yes	Yes	0	0	24	0	1	0
		Newtoniellidae	*Ataxocerithium*		Yes	Yes	10	0	0	5	14	0
		Potamididae			Yes	Yes	1	0	0	0	0	0
	Cephalaspidea	Aglajidae			Yes	Yes	0	0	1	0	0	2
	Cycloneritimorpha				Yes	Yes	0	0	0	2	0	0
		Neritidae			Yes	Yes	3	0	0	0	0	0
	Littorinimorpha				Yes	Yes	0	0	0	9	3	0
		Bursidae			Yes	Yes	1	0	0	0	0	0
			Bursina		Yes	No	1	0	0	0	0	0
		Calyptraeidae			Yes	Yes	1	0	0	0	0	0
		Cassidae			Yes	Yes	0	0	1	0	0	0
			Semicassis		Yes	Yes	1	0	0	0	0	0

Class	Order	Family	Genus	Species	In Australia [5]	Rottnest [5]	Copepod 3	Cnidaria	Mollusca	Copepod 2	Copepod 1	18S
		Cypraeidae	*Erosaria*	*Erosaria cernica*	Yes	Yes	0	2	0	0	0	0
			Staphylaea	*Staphylaea limacina*	Yes	No	0	0	0	0	1	0
		Hipponicidae	*Antisabia*	*Antisabia foliacea*	Yes	Yes	1	0	4	0	0	0
		Naticidae	*Conuber*		Yes	Yes	0	0	0	0	1	0
		Rissoinidae	*Phosinella*	*Phosinella clathrata*	Yes	No	0	0	0	0	0	1
		Strombidae			Yes	Yes	0	0	0	1	0	0
			Canarium	*Canarium mutabile*	Yes	Yes	0	0	0	0	1	0
		Triviidae	*Ellatrivia*	*Ellatrivia merces*	Yes	Yes	0	0	0	0	1	0
	Neogastropoda				Yes	Yes	0	0	1	2	6	1
		Buccinidae			Yes	Yes	2	0	2	0	0	0
			Lirabuccinum		No	No	8	0	0	0	0	0
				Lirabuccinum dirum	No	No	1	0	0	0	0	0
		Columbellidae			Yes	Yes	1	0	2	0	0	0
		Conidae			Yes	Yes	1	0	0	0	0	0
			Conus	*Conus*	Yes	Yes	0	0	0	0	2	0
				Conus klemae	Yes	Yes	1	0	1	0	2	0
		Mitridae	*Mitra*	*Mitra cucumerina*	Yes	No	0	0	0	0	1	0
		Muricidae			Yes	Yes	23	0	1	1	1	0
			Cronia		Yes	Yes	0	0	1	2	0	0
			Dicathais	*Dicathais orbita*	Yes	Yes	0	0	2	0	3	0
	Nudibranchia				Yes	Yes	0	10	2	1	2	0
		Chromodorididae			Yes	Yes	0	1	0	0	0	0
			Ceratosoma	*Ceratosoma amoenum*	Yes	Yes	0	2	1	0	0	0
			Chromodoris	*Chromodoris striatella*	Yes	No	0	6	1	0	0	0
		Dotidae	*Doto*		Yes	Yes	0	0	1	0	0	0
		Eubranchidae			Yes	No	0	0	0	0	1	0
		Tergipedidae			Yes	Yes	0	0	0	0	2	0
	Pleurobranchomorpha	Pleurobranchidae			Yes	Yes	0	0	1	0	0	0
			Pleurobranchus	*Pleurobranchus hilli*	Yes	Yes	0	3	0	0	0	0

Class	Order	Family	Genus	Species	In Australia [5]	Rottnest [5]	Copepod 3	Cnidaria	Mollusca	Copepod 2	Copepod 1	18S
	Sacoglossa				Yes	Yes	0	1	0	0	0	0
		Limapontiidae			Yes	Yes	0	0	1	0	0	0
	Thecosomata				Yes	Yes	0	1	1	0	0	0
		Creseidae			Yes	No	0	0	11	0	0	0
			Creseis		Yes	No	0	1	18	0	0	4
				Creseis clava	No	No	0	0	1	0	0	0
		Cuvierinidae	*Cuvierina*		Yes	Yes	0	1	0	0	0	0
Polyplacophora	Chitonida	Mopaliidae			Yes	Yes	2	0	0	0	0	0
