# Supplementary material for: Marine environmental DNA biomonitoring reveals seasonal patterns in biodiversity and identifies ecosystem responses to anomalous climatic events
Source: PLoS Genet. 2019 Feb 8;15(2):e1007943. doi: 10.1371/journal.pgen.1007943 (PMC6368286; doi:10.1371/journal.pgen.1007943)
Supplement: S5 Table — (PDF) [file pgen.1007943.s005.pdf]

Table S5: Number of Cnidaria detections in Rottnest Island zooplankton samples by each assay.

Class	Order	Family	Genus	Species	In Australia [5]	Rottnest [5]	Copepod 3	Cnidaria	Crust	Mollusca	18S
Anthozoa	Actiniaria				Yes	Yes	3	0	0	2	1
	Pennatulacea	Virgulariidae	*Stylatula*	*Stylatula elongata*	No	No	0	1	0	1	0
	Scleractinia	Caryophylliidae			Yes	Yes	1	0	0	0	0
	Zoantharia				Yes	Yes	1	1	0	3	0
		Zoanthidae			Yes	Yes	0	1	0	1	0
		Microzoanthidae	*Microzoanthus*		Yes	No	0	2	0	0	0
Hydrozoa					Yes	Yes	0	1	0	0	0
	Anthoathecata				Yes	Yes	0	9	0	0	7
		Oceaniidae	*Turritopsis*		Yes	No	0	0	0	11	0
		Pennariidae	*Pennaria*		Yes	Yes	0	1	0	0	0
	Leptothecata				Yes	Yes	0	7	0	0	0
		Campanulariidae			Yes	Yes	0	9	0	1	0
			Clytia		Yes	Yes	0	1	0	4	0
			Obelia		Yes	Yes	0	1	0	0	0
		Eirenidae	*Eirene*		Yes	No	0	0	0	1	0
		Plumulariidae			Yes	Yes	0	0	0	1	0
		Thyroscyphidae	*Thyroscyphus*	*Thyroscyphus ramosus*	No	No	0	0	0	0	1
	Narcomedusae	Cuninidae	*Cunina*		Yes	No	0	0	0	1	0
	Siphonophorae				Yes	Yes	0	3	0	0	2
		Abylidae	*Abylopsis*	*Abylopsis eschscholtzii*	No	No	0	1	0	0	0
			Bassia	*Bassia bassensis*	No	No	0	17	0	0	0
		Agalmatidae			Yes	No	0	2	0	2	0
			Athorybia	*Athorybia rosacea*	No	No	0	1	0	0	0
			Nanomia		Yes	No	0	3	0	0	0
				Nanomia bijuga	No	No	2	0	0	0	1
		Cordagalmatidae	*Cordagalma*	*Cordagalma ordinatum*	No	No	0	0	0	0	1
		Diphyidae			Yes	Yes	0	2	0	0	0
			Lensia		No	No	0	2	0	0	0
				Lensia campanella	No	No	1	0	0	0	0
				Lensia subtiloides	No	No	0	4	0	21	0

Class	Order	Family	Genus	Species	In Australia [5]	Rottnest [5]	Copepod 3	Cnidaria	Crust	Mollusca	18S
		Sphaeronectidae	*Sphaeronectes*	*Sphaeronectes koellikeri*	No	No	0	33	0	4	0
	Trachymedusae	Geryoniidae			Yes	Yes	0	0	1	0	2
			Geryonia	*Geryonia proboscidalis*	Yes	No	1	0	0	0	0
			Liriope		Yes	No	0	2	0	0	0
				Liriope tetraphylla	Yes	No	27	0	0	0	0
		Rhopalonematidae	*Aglaura*	*Aglaura hemistoma*	Yes	No	0	11	0	0	0
Myxozoa	Bivalvulida	Myxidiidae			Yes	No	0	0	0	0	1
Scyphozoa	Semaestomeae	Pelagiidae	*Chrysaora*		Yes	Yes	0	1	0	1	1
