# Supplementary material for: Marine environmental DNA biomonitoring reveals seasonal patterns in biodiversity and identifies ecosystem responses to anomalous climatic events
Source: PLoS Genet. 2019 Feb 8;15(2):e1007943. doi: 10.1371/journal.pgen.1007943 (PMC6368286; doi:10.1371/journal.pgen.1007943)
Supplement: S6 Table — (PDF) [file pgen.1007943.s006.pdf]

Table S6: Number of Echinodermata detections in Rottnest Island zooplankton samples by each assay.

Class	Order	Family	Genus	Species	In Australia [5]	Rottnest [5]	Copepod 3	Cnidaria	Crust	Mollusca	Copepod 2	18S
Asteroidea	Velatida				Yes	Yes	0	1	0	0	0	0
Crinoidea	Comatulida				Yes	Yes	0	2	0	0	0	0
		Antedonidae			Yes	Yes	0	0	0	2	0	0
Echinoidea - Echinacea					Yes	Yes	0	0	0	0	0	1
Echinodea	Camarodonta	Echinometridae	*Echinometra*	*Echinometra mathaei*	Yes	Yes	0	2	0	2	0	0
		Temnopleuridae			Yes	Yes	0	0	0	0	2	1
			Temnopleurus	*Temnopleurus michaelseni*	Yes	Yes	10	12	0	5	5	0
	Clypeasteroida				Yes	Yes	0	3	0	0	0	0
	Spatangoida				Yes	Yes	0	8	0	2	0	0
		Loveniidae			Yes	Yes	0	1	0	0	0	0
Ophiuroidea	Ophiurida				Yes	Yes	0	2	0	0	0	1
		Amphiuridae	*Amphipholis*		Yes	Yes	0	0	2	0	0	0
		Ophionereididae	*Ophionereis*	*Ophionereis schayeri*	Yes	Yes	0	2	0	0	0	0
