# Supplementary material for: Marine environmental DNA biomonitoring reveals seasonal patterns in biodiversity and identifies ecosystem responses to anomalous climatic events
Source: PLoS Genet. 2019 Feb 8;15(2):e1007943. doi: 10.1371/journal.pgen.1007943 (PMC6368286; doi:10.1371/journal.pgen.1007943)
Supplement: S7 Table — (PDF) [file pgen.1007943.s007.pdf]

Table S7: Number of other Animalia taxa detections in Rottnest Island zooplankton samples by each assay.

Phyla	Class	Order	Family	Genus	Species	Australia [5]	Rottnest [5]	Copepod 3	Cnidaria	Crust	Mollusca	Copepod 2	Copepod 1	18S
Annelida	Polychaeta	Echiuroidea	Bonelliidae	*Bonellia*		Yes	Yes	0	0	0	1	0	0	0
		Phyllodocida				Yes	Yes	1	2	0	3	0	0	1
		Spionida	Spionidae			Yes	Yes	0	0	0	0	0	0	2
Bryozoa	Gymnolaemata	Cheilostomata	Candidae	*Tricellaria (ALA)*		Yes	No	0	0	1	0	0	0	0
			Membraniporidae	*Biflustra*		Yes	Yes	0	0	0	2	0	0	0
			Watersiporidae	*Watersipora*	*Watersipora cucullata*	Yes	No	0	0	0	2	0	0	0
		Ctenostomatida	Flustrellidridae	*Flustrellidra*	*Flustrellidra armata*	No	No	1	0	0	0	1	2	0
Chaetognatha	Sagittoidea	Aphragmophora	Sagittidae			Yes	Yes	0	1	0	0	0	0	7
				Aidanosagitta	*Aidanosagitta neglecta*	Yes	Yes	0	0	0	0	0	0	2
				Sagitta		Yes	Yes	1	0	0	2	0	2	0
					Sagitta bipunctata	Yes	Yes	0	0	0	0	0	2	0
Ctenophora	Nuda	Berioda	Beroidae	*Beroe*		Yes	No	0	0	0	0	0	0	1
Porifera	Demospongiae					Yes	Yes	0	1	0	0	0	0	0
		Axinellida	Axinellidae			Yes	Yes	0	1	0	0	0	0	0
		Bubarida	Dictyonellidae	*Acanthella*		Yes	Yes	0	1	0	0	0	0	0
		Clionaida	Clionaidae	*Cliona*	*Cliona jullieni*	No	No	0	0	0	3	0	0	0
		Dendroceratida	Darwinellidae	*Dendrilla*		Yes	Yes	1	0	0	0	0	0	0
		Poecilosclerida				Yes	Yes	1	1	0	1	0	0	1
			Hymedesmiidae	*Phorbas*		Yes	Yes	0	0	0	1	0	0	0
			Microcionidae	*Clathria*		Yes	Yes	0	0	0	1	0	0	0
				Ophlitaspongia	*Ophlitaspongia papilla*	No	No	0	0	0	1	0	0	0
		Tetractinellida	Ancorinidae	*Stelletta*		Yes	Yes	0	1	0	0	0	0	0
				Stellettinopsis	*Stellettinopsis megastylifera*	No	No	0	1	0	0	0	0	0
			Geodiinae	*Geodia*		Yes	Yes	0	1	0	0	0	0	0
Nemertea	Anopla	Paleonemertea				No	No	0	0	0	0	0	1	0
			Lineidae			Yes	No	3	0	0	0	0	0	0
	Enopla	Monostilifera	Amphiporidae			Yes	No	1	0	0	0	0	0	0
		Polystilifera	Paradrepanophoridae			No	No	0	2	0	0	0	0	0
	Paleonemertea					No	No	0	0	0	0	0	1	0
			Cephalothricidae	*Cephalothrix*	*Cephalothrix filiformis*	No	No	0	0	0	0	0	0	1
Platyhelminthes	Rhabditophora	Polycladida				Yes	Yes	0	8	0	1	0	0	0
	Trematoda	Plagiorchiida	Didymozoidae	*Didymozoon*		No	No	0	0	0	0	0	0	1

Phyla	Class	Order	Family	Genus	Species	Australia [5]	Rottnest [5]	Copepod 3	Cnidaria	Crust	Mollusca	Copepod 2	Copepod 1	18S
Sipuncula	Phascolosomatidea	Phascolosomatida	Phascolosomatidae	*Apionsoma*	*Apionsoma (Apionsoma) misakianum*	Yes	No	0	0	0	0	0	0	1
