# Supplementary material for: Marine environmental DNA biomonitoring reveals seasonal patterns in biodiversity and identifies ecosystem responses to anomalous climatic events
Source: PLoS Genet. 2019 Feb 8;15(2):e1007943. doi: 10.1371/journal.pgen.1007943 (PMC6368286; doi:10.1371/journal.pgen.1007943)
Supplement: S8 Table — (PDF) [file pgen.1007943.s008.pdf]

Table S8: Pairwise analysis of seasonal OTU richness & assemblage, t statistics included for significant results (t)—PERMANOVA+[7].

Assay	OTU diversity test	Summer Spring	Summer Winter	Summer Autumn	Spring Winter	Spring Autumn	Winter Autumn
Cnidaria	Richness	-	-	-	-	-	-
	Assemblage	* HB t=1.34	*** HB t=2.02	** HB t=1.45	*** HB t=1.62	*** HB t=1.55	** HB t=1.40
Copepod 1	Richness	-	-	-	-	-	-
	Assemblage	* t=1.28	*** HB t=1.85	*** HB t= 1.69	** HB t=1.61	*** HB t=1.71	-
Copepod 2	Richness	-	* t=2.48	-	-	-	-
	Assemblage	-	*** HB t=2.10	-	*** HB t=1.95	** HB t=1.61	-
Copepod 3	Richness	-	-	-	-	-	-
	Assemblage	* t=1.35	*** HB t=2.13	* HB t=1.53	*** HB t=2.12	*** HB t=1.90	-
Crustacea	Richness	* t=2.13	-	-	* =1.76	-	-
	Assemblage	** HB t=1.49	*** HB t=1.76	* HB t=1.40	** HB t=1.54	*** HB t=1.60	* HB t=1.36
Fish	Richness	-	*** HB t =5.03	*** HB t= 3.89	*** HB t= 4.18	** HB t= 3.25	-
	Assemblage	** HB t =1.61	*** HB t =2.20	** HB t =1.73	*** HB t= 2.43	*** HB t =2.23	*** HB t=2.02
Mollusca	Richness	-	-	-	-	-	-
	Assemblage	* HB t=1.31	*** HB t=2.37	** HB t=1.55	*** HB t=1.93	*** HB t=1.55	*** HB t=1.73
Universal	Richness	-	-	-	-	-	-
	Assemblage	* t=1.41	*** HB t=1.87	* t=1.54	** HB t=1.59	* t=1.43	* t=1.40

Where *** is $p \leq .001$, ** is $p \leq .01$, * is $p \leq .05$, HB is significant with Holm-Bonferroni correction & - is no significant changes
