# Supplementary material for: Marine environmental DNA biomonitoring reveals seasonal patterns in biodiversity and identifies ecosystem responses to anomalous climatic events
Source: PLoS Genet. 2019 Feb 8;15(2):e1007943. doi: 10.1371/journal.pgen.1007943 (PMC6368286; doi:10.1371/journal.pgen.1007943)
Supplement: S9 Table — (PDF) [file pgen.1007943.s009.pdf]

Table S9: Indicator species analysis for seasonal variation—*Indval* [8]

Season	Assay	OTU	Taxa	Indicator value	*p* value
Spring	Fish	6	Labridae (two – 96%)	0.6400	0.001
Spring	Universal	37	Hexanauplia	0.4139	0.001
Spring	Copepod 3	125	Perciformes	0.3483	0.004
Spring	Cnidaria	152	Plantae	0.3217	0.007
Spring	Copepod 3	242	*Clausocalanus pergens* (v)	0.3195	0.017
Spring	Fish	12	*Austrolabrus maculatus*	0.3176	0.012
Spring	Copepod 3	186	*Bestiolina sp*	0.3150	0.020
Spring	Mollusca	43	Mullidae	0.3148	0.008
Spring	Copepod 2	40	Hexanauplia	0.3006	0.014
Spring	Copepod 3	115	Paracalanidae	0.2950	0.011
Spring	Cnidaria	18	Arthropoda	0.2948	0.045
Spring	Fish	11	*Upeneichthys stotti*	0.2904	0.017
Spring	Copepod 3	2	*Bestiolina sp.*	0.2833	0.025
Spring	Copepod 3	166	*Bestiolina sp.*	0.2816	0.017
Spring	Copepod 1	82	*Dicathais orbita* (v)	0.2708	0.011
Spring	Copepod 3	303	Labridae	0.2667	0.005
Spring	Cnidaria	38	Leptothecata	0.2667	0.010
Spring	Copepod 3	128	*Evadne spinifera* (v)	0.2659	0.038
Spring	Cnidaria	37	Echinoidea	0.2430	0.043
Spring	Mollusca	202	Animalia	0.2299	0.022
Spring	Mollusca	251	Anopla	0.2286	0.033
Spring	Copepod 1	41	Gastropoda	0.2133	0.026
Spring	Crustacea	36	Arthropoda	0.2083	0.033
Spring	Mollusca	245	*Centropages violaceus* (v)	0.2000	0.048
Spring	Cnidaria	214	Phyllodocida.	0.2000	0.050
Spring	Cnidaria	69	Cnidaria	0.1778	0.047
Summer	Copepod 3	22	*Calcinus dapsiles*	0.6661	0.001
Summer	Crustacea	16	*Calcinus dapsiles*	0.4952	0.001
Summer	Cnidaria	13	*Evadne spinifera* (v)	0.4535	0.001
Summer	Mollusca	3	*Evadne spinifera* (v)	0.4258	0.001
Summer	Copepod 3	296	Calanidae	0.4000	0.002
Summer	Cnidaria	17	*Etrumeus jacksoniensis*	0.3786	0.001
Summer	Mollusca	93	Arthropoda	0.3752	0.004
Summer	Cnidaria	34	Chlorophyta	0.3663	0.004
Summer	Mollusca	88	Malacostraca	0.3661	0.003
Summer	Mollusca	109	Malacostraca	0.3607	0.002
Summer	Cnidaria	91	*Chromodoris sp.*	0.3510	0.004
Summer	Copepod 1	109	*Triconia sp.*	0.3437	0.005
Summer	Mollusca	263	*Chromodoris striatella* (v)	0.3355	0.006
Summer	Fish	37	*Chromis sp.*	0.3333	0.004
Summer	Cnidaria	15	Arthropoda	0.3298	0.016
Summer	Cnidaria	10	Animalia	0.3283	0.006
Summer	Mollusca	257	Animalia	0.3242	0.009
Summer	Copepod 3	194	Arthropoda	0.3169	0.006
Summer	Copepod 1	34	*Triconia sp.*	0.3169	0.007
Summer	Cnidaria	14	Hydrozoa	0.3029	0.033
Summer	Mollusca	179	Hexanauplia	0.2889	0.009

Season	Assay	OTU	Taxa	Indicator value	*p* value
Summer	Mollusca	184	Hydrozoa	0.2877	0.024
Summer	Mollusca	247	*Trachurus sp.* (Two at 100% (v)).	0.2778	0.015
Summer	Mollusca	97	Podonidae	0.2751	0.036
Summer	Mollusca	106	Rhodophyta	0.2716	0.019
Summer	Mollusca	5	*Cliona jullieni*	0.2708	0.012
Summer	Cnidaria	86	Alveolata	0.2683	0.033
Summer	Mollusca	54	Biemnidae (Two at 100% (v))	0.2667	0.012
Summer	Mollusca	343	Arthropoda	0.2667	0.017
Summer	Mollusca	292	Arthropoda	0.2595	0.016
Summer	Universal	15	Eukaryota	0.2579	0.015
Summer	Mollusca	137	Calanoida	0.2553	0.037
Summer	Mollusca	267	Malacostraca	0.2537	0.016
Summer	Crustacea	45	Animalia	0.2537	0.026
Summer	Cnidaria	135	Chlorophyta	0.2537	0.031
Summer	Cnidaria	71	Arthropoda	0.2483	0.042
Summer	Copepod 3	247	*Actinaria* sp. (v)	0.2328	0.022
Summer	Mollusca	84	Animalia	0.2307	0.031
Summer	Mollusca	281	Arthropoda	0.2307	0.037
Summer	Mollusca	20	Sapphirinidae	0.2299	0.028
Summer	Copepod 3	176	*Chromis sp.*	0.2133	0.028
Summer	Cnidaria	46	*Pleurobranchus hilli*	0.2133	0.029
Summer	Mollusca	108	Annelida	0.2133	0.031
Summer	Copepod 3	59	*Chromis notata*	0.2070	0.045
Summer	Cnidaria	167	Gastropoda	0.2044	0.039
Summer	Fish	13	*Chromis notata*	0.2032	0.044
Summer	Universal	87	Chromista	0.1778	0.045
Autumn	Mollusca	85	*Farranula gibbula* (v)	0.3858	0.002
Autumn	Copepod 3	55	*Flaccisagitta enflata*	0.3844	0.001
Autumn	Copepod 3	90	*Centropages orsinii*	0.3757	0.002
Autumn	Fish	3	*Engraulis sp.* (Three at 100%)	0.3630	0.003
Autumn	Universal	79	Sagittidae	0.3611	0.003
Autumn	Cnidaria	30	*Temnopleurus michaelseni*	0.3528	0.010
Autumn	Crustacea	63	Arthropoda	0.35	0.007
Autumn	Crustacea	34	Animalia	0.3362	0.015
Autumn	Copepod 1	122	*Undinula vulgaris* (v)	0.3326	0.005
Autumn	Copepod 1	37	*Clausocalanus minor*	0.3313	0.005
Autumn	Cnidaria	61	Prasinophyceae	0.3297	0.034
Autumn	Copepod 3	293	Bivalvia	0.3278	0.002
Autumn	Crustacea	30	Animalia	0.3278	0.003
Autumn	Cnidaria	184	Ophiuroidea	0.3278	0.005
Autumn	Crustacea	35	Animalia	0.3278	0.007
Autumn	Universal	23	Eukaryota	0.3248	0.020
Autumn	Copepod 3	122	*Calocalanus pavo* (v)	0.3159	0.016
Autumn	Copepod 3	290	Malacostraca	0.3077	0.005
Autumn	Copepod 3	104	Sagittidae	0.3074	0.021
Autumn	Mollusca	33	Decapoda	0.3074	0.040
Autumn	Copepod 3	216	Copepoda	0.3036	0.013
Autumn	Cnidaria	117	Animalia	0.3033	0.019
Autumn	Copepod 1	97	Caenogastropoda	0.2984	0.014

Season	Assay	OTU	Taxa	Indicator value	*p* value
Autumn	Universal	42	Sargassaceae (Many at 100%)	0.2984	0.022
Autumn	Copepod 1	125	*Acrocalanus gracilis* (v)	0.2979	0.032
Autumn	Copepod 3	139	*Paracalanus sp.* (Two at 100%)	0.2838	0.020
Autumn	Copepod 3	266	Ophiurida	0.2797	0.014
Autumn	Crustacea	74	Animalia	0.2767	0.010
Autumn	Copepod 3	80	*Temnopleurus michaelseni*	0.2751	0.044
Autumn	Mollusca	45	Animalia	0.2740	0.038
Autumn	Mollusca	28	Animalia	0.2705	0.026
Autumn	Cnidaria	23	Hydrozoa	0.2530	0.022
Autumn	Copepod 3	297	Euralida	0.2530	0.025
Autumn	Copepod 2	66	*Centropages orsinii*	0.2529	0.012
Autumn	Crustacea	12	Eukaryota	0.2510	0.048
Autumn	Mollusca	259	Annelida	0.2473	0.034
Autumn	Copepod 2	80	*Acrocalanus sp.*	0.2412	0.047
Autumn	Copepod 3	188	*Sagitta sp.*	0.2331	0.034
Autumn	Crustacea	9	*Menaethius sp.*	0.2069	0.042
Winter	Mollusca	68	*Canthocalanus pauper*	0.4987	0.001
Winter	Copepod 3	110	*Centropages furcatus*	0.4952	0.001
Winter	Mollusca	158	Poecilostomatoida	0.4710	0.001
Winter	Copepod 2	19	Hexanauplia	0.4646	0.001
Winter	Mollusca	78	Calanoida	0.4447	0.003
Winter	Cnidaria	90	Gastropoda	0.4288	0.002
Winter	Mollusca	174	Arthropoda	0.4183	0.004
Winter	Mollusca	38	*Eucalanus pseudoattenuatus* (v)	0.4148	0.003
Winter	Copepod 1	11	*Eucalanus pseudattenuatus* (v)	0.4088	0.002
Winter	Mollusca	180	Arthropoda	0.4050	0.001
Winter	Copepod 2	18	Hexanauplia	0.3985	0.004
Winter	Copepod 2	46	Crustacea	0.3976	0.002
Winter	Mollusca	47	*Subeucalanus pileatus* (v)	0.3965	0.005
Winter	Copepod 2	55	*Paracalanus sp.* (Two at 100%(v))	0.3908	0.005
Winter	Universal	91	Pycnococcaceae (Many at 100%)	0.3852	0.003
Winter	Copepod 3	93	*Paracalanus sp.* (Two at 100%(v))	0.3824	0.002
Winter	Mollusca	107	Calanoida	0.3779	0.007
Winter	Copepod 3	121	Sagittidae	0.3777	0.008
Winter	Copepod 3	83	*Acrocalanus gracilis*	0.3692	0.007
Winter	Mollusca	130	*Ecklonia radiata*	0.3685	0.005
Winter	Universal	52	*Leptochela sp*	0.3592	0.001
Winter	Copepod 3	138	Calanoida	0.3552	0.005
Winter	Copepod 2	5	*Lucicutia flavicornis* (v)	0.3540	0.009
Winter	Copepod 2	44	*Eucalanus pseudattenuatus* (v)	0.3504	0.014
Winter	Copepod 3	73	Calanoida	0.3486	0.012
Winter	Copepod 3	58	*Flaccisagitta enflata*	0.3394	0.021
Winter	Mollusca	144	*Paracalanus sp.* (Two at 100% (v))	0.3380	0.033
Winter	Copepod 2	32	*Paracalanus sp.*	0.3373	0.014
Winter	Mollusca	79	*Calocalanus pavo* (v)	0.3364	0.013
Winter	Copepod 3	107	Calanoida	0.3346	0.006
Winter	Cnidaria	12	*Bassia bassensis*	0.3283	0.008
Winter	Copepod 2	7	*Delibus sp.*	0.3283	0.043
Winter	Copepod 3	37	*Undinula vulgaris* (v)	0.3267	0.024

Season	Assay	OTU	Taxa	Indicator value	*p* value
Winter	Copepod 2	35	Paracalanidae	0.3267	0.026
Winter	Mollusca	110	Animalia	0.3251	0.025
Winter	Copepod 3	14	*Cosmocalanus darwinii*	0.3223	0.047
Winter	Copepod 2	16	*Clausocalanus minor*	0.3221	0.048
Winter	Mollusca	116	Arthropoda	0.3209	0.008
Winter	Copepod 3	85	*Canthocalanus pauper*	0.3205	0.029
Winter	Cnidaria	45	Animalia	0.3183	0.022
Winter	Copepod 3	200	Arthropoda	0.3176	0.007
Winter	Copepod 3	215	*Euchaeta concinna* (v)	0.3176	0.007
Winter	Copepod 3	52	*Eucalanus pseudattenuatus* (v)	0.3168	0.030
Winter	Copepod 3	274	*Subeucalanus pileatus* (v)	0.3157	0.007
Winter	Copepod 2	95	*Scolecithricella longispinosa* (v)	0.3099	0.006
Winter	Copepod 3	64	*Sagitta sp.*	0.3074	0.040
Winter	Copepod 3	78	*Flaccisagitta enflata*	0.3074	0.048
Winter	Copepod 3	79	*Scolecithrix danae* (v)	0.3070	0.034
Winter	Mollusca	220	Calanoida (Two at 98% (v))	0.3062	0.024
Winter	Copepod 1	69	*Paracalanus sp.*	0.3062	0.024
Winter	Copepod 3	145	Arthropoda	0.3011	0.046
Winter	Copepod 3	50	*Paracalanus aculeatus*	0.3011	0.048
Winter	Cnidaria	96	Animalia	0.2995	0.045
Winter	Copepod 1	12	Arthropoda	0.2953	0.048
Winter	Cnidaria	112	*Pontellina plumata* (v)	0.2928	0.007
Winter	Mollusca	51	*Lucicutia flavicornis* (v)	0.2923	0.039
Winter	Mollusca	50	Hexanauplia	0.2913	0.028
Winter	Copepod 3	111	*Lucifer intermedius*	0.2893	0.015
Winter	Copepod 3	189	*Sagitta sp.*	0.2888	0.018
Winter	Copepod 3	217	Arthropoda	0.2778	0.009
Winter	Copepod 2	53	Calanoida	0.2778	0.009
Winter	Copepod 2	84	Palaeonemertea	0.2778	0.010
Winter	Copepod 3	101	*Cosmocalanus sp.*	0.2756	0.026
Winter	Copepod 3	244	Arthropoda	0.2725	0.018
Winter	Copepod 3	282	Annelida	0.2708	0.011
Winter	Mollusca	17	*Lucifer sp.* (v)	0.2686	0.020
Winter	Mollusca	132	Animalia	0.2678	0.033
Winter	Crustacea	31	Animalia	0.2657	0.023
Winter	Mollusca	171	Animalia	0.2657	0.046
Winter	Copepod 3	208	Arthropoda	0.2586	0.023
Winter	Copepod 3	196	*Flaccisagitta enflata*	0.2574	0.045
Winter	Copepod 1	76	*Calocalanus pavo* (v)	0.2574	0.047
Winter	Copepod 1	32	*Animalia*	0.2500	0.003
Winter	Copepod 3	113	Valvatida	0.2500	0.006
Winter	Crust 16S	125	Animalia	0.2500	0.006
Winter	Copepod 3	65	Arthropoda	0.2500	0.012
Winter	Copepod 2	31	Hexanauplia	0.2481	0.047
Winter	Copepod 1	148	*Calanoida*	0.2397	0.035
Winter	Crustacea	111	Malacostraca	0.2381	0.017
Winter	Copepod 1	28	Calanidae	0.2327	0.049
Winter	Cnidaria	89	Asteroidea	0.2126	0.040
Winter	Mollusca	321	Arthropoda	0.1912	0.037

Season	Assay	OTU	Taxa	Indicator value	*p* value
Winter	Copepod 3	261	Anthoathecata	0.1912	0.039
Winter	Cnidaria	148	*Sagitta sp.*	0.1912	0.049
Winter	Crustacea	60	Arthropoda	0.1667	0.036
Winter	Copepod 3	182	Ophiurida	0.1667	0.044
Winter	Cnidaria	238	Cidaridae	0.1667	0.045
Winter	Copepod 2	72	Animalia	0.1667	0.049
Winter	Copepod 1	154	*Arthropoda*	0.1667	0.050

(v) Matched to vouchered specimen sequence
