# Supplementary material for: Marine environmental DNA biomonitoring reveals seasonal patterns in biodiversity and identifies ecosystem responses to anomalous climatic events
Source: PLoS Genet. 2019 Feb 8;15(2):e1007943. doi: 10.1371/journal.pgen.1007943 (PMC6368286; doi:10.1371/journal.pgen.1007943)
Supplement: S10 Table — (PDF) [file pgen.1007943.s010.pdf]

Table S10: Pairwise analysis of yearly OTU richness & assemblage, t statistics included for significant results (t)—PERMANOVA+[7].

Assay	OTU diversity test	2010 2011	2010 2012	2010 2013	2010 2014	2011 2012	2011 2013	2011 2014	2012 2013	2012 2014	2013 2014
Cnidaria	Richness	-	-	-	-	-	-	-	-	-	-
	Assemblage	-	*** HB t=1.63	*** HB t=1.81	*** HB t=2.01	* t=1.33	** HB t=1.49	** HB t=1.51	-	-	-
Copepod 1	Richness	-	* t=2.89	-	* t=2.99	** HB t=3.81	* t=2.67	*** HB t=4.10	-	-	-
	Assemblage	* t=1.38	*** HB t=1.96	*** HB t=1.85	*** HB t=2.33	** HB t=1.73	** HB t=1.57	*** HB t=2.07	-	* t=1.28	* t =1.32
Copepod 2	Richness	-	* t=2.73	-	-	-	-	-	-	-	-
	Assemblage	* t=1.37	** t=1.51	-	-	* t=1.45	-	* t=1.37	-	* t=1.34	-
Copepod 3	Richness	* t=2.50	** HB t= 3.61	** t=3.04	* t=2.29	* t=2.23	-	-	-	-	-
	Assemblage	* t=1.32	** HB t=1.71	** HB t=1.48	* t=1.40	** t=1.49	* t=1.37	* t=1.47	-	-	-
Crustacea	Richness	* t=2.58	* t= 2.14	-	-	-	-	-	-	-	-
	Assemblage	-	-	-	-	-	-	-	-	** t=1.45	-
Fish	Richness	-	-	-	-	-	-	-	-	-	-
	Assemblage	-	-	-	-	-	-	-	-	-	-
Mollusca	Richness	-	-	-	-	-	-	-	-	-	-
	Assemblage	* t=1.30	*** HB t=1.73	*** HB t=1.57	** HB t=1.65	** HB t=1.48	** HB t =1.43	** HB t =1.56	-	-	-
Universal	Richness	-	-	-	-	-	-	-	-	-	-
	Assemblage	-	* t=1.34	** HB t=1.46	*** HB t= 1.67	-	-	** t =1.86	-	-	-

Where *** is $p \leq .001$, ** is $p \leq .01$, * is $p \leq .05$, ^{HB} is significant with Holm-Bonferroni correction & - is no significant changes
