# Supplementary material for: Marine environmental DNA biomonitoring reveals seasonal patterns in biodiversity and identifies ecosystem responses to anomalous climatic events
Source: PLoS Genet. 2019 Feb 8;15(2):e1007943. doi: 10.1371/journal.pgen.1007943 (PMC6368286; doi:10.1371/journal.pgen.1007943)
Supplement: S11 Table — (PDF) [file pgen.1007943.s011.pdf]

Table S11: Indicator species analysis for yearly variation —*Indval* [8]

Year	Assay	OTU	Taxa	Indicator value	*p* value
2010	Cnidaria	165	Pythiales	0.6667	0.001
2010	Cnidaria	175	Animalia	0.4540	0.001
2010	Mollusca	216	Arthropoda	0.4444	0.001
2010	Copepod 2	11	Arthropoda	0.4444	0.001
2010	Copepod 1	57	Arthropoda	0.4123	0.001
2010	Mollusca	36	*Creseis sp.*	0.4085	0.002
2010	Mollusca	333	*Oncaea venusta typica*	0.3612	0.008
2010	Mollusca	137	Calanoida	0.3341	0.004
2010	Crustacea	24	Xanthidae	0.3333	0.003
2010	Cnidaria	21	Hydrozoa	0.3325	0.012
2010	Copepod 1	74	*Paracalanus Indicus*	0.3320	0.014
2010	Mollusca	118	Drepanophoridae	0.3099	0.027
2010	Cnidaria	95	Oithonidae	0.3045	0.016
2010	Cnidaria	54	*Acartia negligens*	0.2941	0.035
2010	Cnidaria	24	*Aglaura hemistoma*	0.2911	0.022
2010	Cnidaria	22	*Penilia avirostris*	0.2910	0.050
2010	Cnidaria	72	Thalassiosirales	0.2736	0.030
2010	Fish	9	Perciformes	0.2500	0.023
2010	Copepod 3	285	Calanoida	0.2222	0.026
2010	Copepod 3	280	Hexanauplia	0.2222	0.031
2010	Mollusca	252	Polychaeta	0.2222	0.032
2010	Copepod 1	110	Gastropoda	0.2222	0.032
2010	Copepod 1	133	Gastropoda	0.2222	0.032
2010	Universal	78	*Centropages sp.*	0.2222	0.036
2011	Mollusca	17	*Lucifer sp.*	0.4760	0.002
2011	Copepod 3	111	*Lucifer intermedius*	0.3969	0.002
2011	Crustacea	31	Animalia	0.3645	0.004
2011	Cnidaria	105	Hydrozoa	0.3636	0.010
2011	Mollusca	280	*Undinula vulgaris*	0.3240	0.005
2011	Universal	18	Decapoda	0.2978	0.018
2011	Cnidaria	45	Animalia	0.2968	0.026
2011	Copepod 2	107	Mollusca	0.2909	0.016
2011	Crustacea	26	*Thalamita admete*	0.2852	0.012
2011	Universal	33	Heterobranchia	0.2727	0.025
2011	Copepod 3	271	Sagittoidea	0.2727	0.029
2011	Cnidaria	125	Plantae	0.2680	0.045
2011	Universal	38	Palaemonoidea	0.2630	0.032
2011	Universal	71	*Temora sp.*	0.2625	0.050
2011	Copepod 3	85	*Canthocalanus pauper*	0.2583	0.045
2011	Cnidaria	98	Animalia	0.2528	0.049
2011	Universal	61	Syndiniales	0.2424	0.024
2011	Copepod 3	300	*Clausocalanus sp.*	0.2385	0.039
2011	Universal	87	Chromista	0.2385	0.044
2011	Mollusca	8	*Candacia bradyi*	0.2286	0.007
2011	Mollusca	14	*Delibus sp.*	0.2234	0.014
2012	Mollusca	99	Arthropoda	0.4069	0.001
2012	Copepod 3	49	*Lucifer intermedius*	0.4040	0.001

Year	Assay	OTU	Taxa	Indicator value	*p* value
2012	Copepod 2	71	Calanoida	0.3997	0.001
2012	Copepod 3	154	*Paracalanus sp.*	0.3707	0.006
2012	Mollusca	75	Arthropoda	0.3378	0.003
2012	Copepod 3	181	Mollusca	0.3349	0.007
2012	Mollusca	96	Animalia	0.3344	0.009
2012	Cnidaria	16	Chlorophyta	0.3235	0.001
2012	Copepod 3	158	*Calocalanus sp.*	0.3228	0.006
2012	Mollusca	192	Calanoida	0.3223	0.011
2012	Mollusca	4	Eukaryota	0.3152	0.001
2012	Copepod 1	22	Paracalanidae	0.3091	0.014
2012	Mollusca	73	Malacostraca	0.3075	0.017
2012	Copepod 2	61	Calanoida	0.3046	0.022
2012	Copepod 3	86	*Calocalanus styliremis*	0.2972	0.020
2012	Copepod 3	89	Arthropoda	0.2968	0.022
2012	Copepod 3	74	*Lirabuccinum sp.*	0.2911	0.016
2012	Mollusca	134	Gastropoda	0.2909	0.021
2012	Copepod 1	36	Arthropoda	0.2868	0.013
2012	Crustacea	102	Eukaryota	0.2852	0.012
2012	Copepod 3	202	Arthropoda	0.2847	0.038
2012	Copepod 1	81	Arthropoda	0.2840	0.016
2012	Copepod 1	4	*Oncaea waldemari*	0.2758	0.026
2012	Copepod 3	301	Potamididae	0.2731	0.022
2012	Copepod 3	321	Arthropoda	0.2727	0.030
2012	Copepod 3	20	Arthropoda	0.2727	0.032
2012	Copepod 3	237	*Paracalanus nanus*	0.2683	0.018
2012	Cnidaria	228	Animalia	0.2676	0.050
2012	Copepod 3	33	Calanoida	0.2653	0.001
2012	Copepod 3	63	*Ditrichocorycaeus sp.*	0.2565	0.018
2012	Copepod 2	49	*Paracalanus sp.*	0.2562	0.038
2012	Copepod 2	98	Mollusca	0.2525	0.025
2012	Crustacea	97	Animalia	0.2438	0.019
2012	Copepod 3	250	Calanoida	0.2424	0.028
2012	Copepod 1	68	Arthropoda	0.2424	0.032
2012	Copepod 3	293	Bivalvia	0.2385	0.041
2012	Copepod 3	332	*Paracalanus sp.*	0.2285	0.050
2013	Cnidaria	5	Prasinophyceae	0.4231	0.001
2013	Copepod 1	56	Arthropoda	0.4000	0.003
2013	Copepod 1	19	Arthropoda	0.3143	0.013
2013	Mollusca	259	Annelida	0.3088	0.007
2013	Mollusca	63	Mollusca	0.3046	0.023
2013	Universal	13	Chlorophyta	0.3037	0.002
2013	Cnidaria	154	Gastropoda	0.3000	0.012
2013	Cnidaria	38	Leptothecata	0.3000	0.013
2013	Cnidaria	131	Animalia	0.3000	0.014
2013	Cnidaria	30	*Temnopleurus michaelseni*	0.2969	0.031
2013	Copepod 3	25	*Labidocera minuta*	0.2903	0.016
2013	Copepod 2	1	Mollusca	0.2886	0.029
2013	Mollusca	30	*Cacozeliana granarium*	0.2843	0.045
2013	Copepod 3	43	Paracalanidae	0.2829	0.035

Year	Assay	OTU	Taxa	Indicator value	*p* value
2013	Cnidaria	50	*Sagitta sp.*	0.2826	0.035
2013	Cnidaria	199	Nudibranchia	0.2750	0.010
2013	Mollusca	5	*Cliona jullieni*	0.2750	0.020
2013	Copepod 3	26	Arthropoda	0.2694	0.043
2013	Cnidaria	99	Nudibranchia	0.2676	0.036
2013	Universal	79	Sagittidae	0.2672	0.031
2013	Universal	41	Cypridinidae	0.2619	0.018
2013	Mollusca	188	Pyramimonadophyceae	0.2571	0.020
2013	Cnidaria	11	Hydrozoa	0.2565	0.021
2013	Copepod 1	84	Arthropoda	0.2538	0.039
2013	Mollusca	309	Mollusca	0.2378	0.031
2013	Copepod 1	106	Animalia	0.2302	0.039
2013	Cnidaria	242	Arthropoda	0.2302	0.042
2014	Copepod 1	44	*Triconia sp.*	0.4046	0.002
2014	Copepod 1	42	Arthropoda	0.3610	0.002
2014	Mollusca	64	*Oncaea sp.*	0.3146	0.007
2014	Copepod 1	54	Arthropoda	0.3006	0.008
2014	Cnidaria	104	Chlorophyta	0.2955	0.011
2014	Copepod 3	66	*Ctenocalanus vanus*	0.2926	0.007
2014	Copepod 1	62	*Oncaea mediterranea* (v)	0.2874	0.022
2014	Copepod 3	217	Arthropoda	0.2852	0.014
2014	Copepod 1	167	Arthropoda	0.2852	0.018
2014	Copepod 3	28	Muricidae	0.2839	0.010
2014	Copepod 1	31	*Ataxocerithium*	0.2780	0.045
2014	Mollusca	27	Calanoida	0.2772	0.011
2014	Crustacea	132	Eukaryota	0.2727	0.024
2014	Crustacea	20	Eukaryota	0.2727	0.034
2014	Copepod 1	32	Animalia	0.2727	0.045
2014	Copepod 1	20	Arthropoda	0.2725	0.029
2014	Mollusca	178	*Paracalanus nanus* (v)	0.2686	0.044
2014	Cnidaria	55	Viridaeplantae	0.2680	0.038
2014	Cnidaria	91	*Chromodoris sp.*	0.2470	0.023
2014	Copepod 1	21	Gastropoda	0.2346	0.039
2014	Copepod 3	39	Calanoida	0.2281	0.034

(v) Matched to vouchered specimen sequence
