# Supplementary material for: Marine environmental DNA biomonitoring reveals seasonal patterns in biodiversity and identifies ecosystem responses to anomalous climatic events
Source: PLoS Genet. 2019 Feb 8;15(2):e1007943. doi: 10.1371/journal.pgen.1007943 (PMC6368286; doi:10.1371/journal.pgen.1007943)
Supplement: S12 Table — (PDF) [file pgen.1007943.s012.pdf]

Table S12: Pairwise analysis of the OTU richness & assemblage between before, during and after the heatwaves, t statistics included for significant results (t)—PERMANOVA+[7].

Assay	OTU diversity test	Heatwave 1 (5 Months)			Heatwave 2 (17 Months)		
		Before-During	Before-After	During-After	Before-During	Before-After	During-After
Cnidaria	Richness	-	-	-	-	-	-
	Assemblage	* HB t=1.27	*** HB t=1.93	** HB t=1.46	* HB t=1.37	*** HB t=2.12	** HB t =1.48
Copepod 1	Richness	-	* HB t=2.46	** HB t=2.88	-	* HB t=2.71	-
	Assemblage	-	*** HB t= 2.19	* t=1.55	** HB t=1.48	*** HB t =2.39	*** HB t =1.75
Copepod 2	Richness	-	-	-	-	-	-
	Assemblage	-	-	-	-	-	* HB t =1.56
Copepod 3	Richness	-	** HB t=2.84	-	** HB t=3.19	* t=2.34	-
	Assemblage	-	** HB t =1.58	-	** HB t=1.55	* HB t =1.48	* HB t=1.51
Crustacea	Richness	* t=2.32	-	-	** HB t= 2.95	-	-
	Assemblage	-	-	-	-	-	-
Fish	Richness	-	-	-	-	-	-
	Assemblage	-	-	* HB t=1.44	-	-	-
Mollusca	Richness	-	-	-	-	-	-
	Assemblage	-	** HB t=1.64	** HB t=1.54	** HB t=1.43	** HB t=1.62	** HB t=1.47
Universal	Richness	-	-	-	-	-	-
	Assemblage	-	** HB t=1.59	* t=1.41	-	** HB t=1.68	** HB t=1.64

Where *** is $p \leq .001$, ** is $p \leq .01$, * is $p \leq .05$, HB is significant with Holm-Bonferroni correction & - is no significant change
