# Supplementary material for: Marine environmental DNA biomonitoring reveals seasonal patterns in biodiversity and identifies ecosystem responses to anomalous climatic events
Source: PLoS Genet. 2019 Feb 8;15(2):e1007943. doi: 10.1371/journal.pgen.1007943 (PMC6368286; doi:10.1371/journal.pgen.1007943)
Supplement: S13 Table — (PDF) [file pgen.1007943.s013.pdf]

Table S13: Indicator species analysis for five-month heatwave variation—*Indval* [8].

Heatwave	Assay	OTU	Taxa	Indicator value	*p* value
Before	Cnidaria	165	Pythiales	0.7778	0.001
Before	Copepod 1	74	*Paracalanus Indicus*	0.6061	0.002
Before	Copepod 2	14	Calanoida	0.4972	0.023
Before	Copepod 1	57	Arthropoda	0.4855	0.018
Before	Copepod 1	13	Paracalanidae	0.4638	0.015
Before	Universal	23	Alveolata	0.4628	0.030
Before	Cnidaria	87	Chlorophyta	0.4098	0.047
Before	Cnidaria	21	Hydrozoa	0.3984	0.030
Before	Copepod 1	114	Arthropoda	0.3644	0.031
Before	Copepod 2	11	Arthropoda	0.3065	0.036
Before	Mollusca	216	Arthropoda	0.3065	0.037
Before	Cnidaria	168	Plantae	0.2908	0.049
Before	Copepod 3	285	Calanoida	0.2222	0.033
Before	Copepod 1	110	Gastropoda	0.2222	0.033
Before	Copepod 1	133	Gastropoda	0.2222	0.035
Before	Mollusca	252	Polychaeta	0.2222	0.037
During	Mollusca	179	Hexanauplia	0.6502	0.001
During	Copepod 3	41	Actinopterygii	0.6052	0.004
During	Mollusca	201	*Euchaeta longicornis*	0.5715	0.002
During	Cnidaria	125	Plantae	0.5426	0.007
During	Copepod 3	95	Arthropoda	0.5426	0.010
During	Fish	5	*Parapriacanthus elongatus*	0.5256	0.017
During	Mollusca	242	*Euphausia hemigibba*	0.5063	0.004
During	Universal	71	*Temora sp.*	0.5055	0.011
During	Mollusca	87	*Copilia mirabilis* (v)	0.4980	0.019
During	Cnidaria	160	Arthropoda	0.4959	0.018
During	Mollusca	37	*Turritopsis sp.* (Two – 98%)	0.4867	0.019
During	Mollusca	57	Arthropoda	0.4862	0.026
During	Copepod 3	130	*Subeucalanus sp.* (Two – 100%(v))	0.4827	0.024
During	Copepod 3	22	*Calcinus dapsiles*	0.4779	0.025
During	Mollusca	145	*Euphasia recurva* (v)	0.4684	0.011
During	Mollusca	280	*Undinula vulgaris*	0.4671	0.009
During	Cnidaria	158	Corycaeidae	0.4647	0.025
During	Cnidaria	11	Hydrozoa	0.4624	0.011
During	Cnidaria	44	Malacostraca	0.4566	0.041
During	Mollusca	76	*Subeucalanus sp.* (two – 99%)	0.4488	0.033
During	Cnidaria	10	Animalia	0.4413	0.049
During	Mollusca	267	Malacostraca	0.4321	0.029
During	Copepod 3	106	Arthropoda	0.4201	0.050
During	Crustacea	88	Animalia	0.4198	0.025
During	Mollusca	186	Eukaryota	0.4000	0.005
During	Mollusca	197	Gastropoda	0.4000	0.008
During	Mollusca	307	Eukaryota	0.4000	0.008
During	Mollusca	7	Parazoanthidae (Many – 99%)	0.4000	0.008
During	Mollusca	224	Gastropoda	0.4000	0.010
During	Mollusca	209	Arthropoda	0.4000	0.010
During	Mollusca	80	Aglajidae	0.4000	0.011

Heatwave	Assay	OTU	Taxa	Indicator value	*p* value
During	Copepod 3	91	*Euphausia recurva* (v)	0.3813	0.040
During	Universal	33	Heterobranchia	0.3770	0.013
During	Mollusca	15	*Watersipora subovoidea*	0.3770	0.013
During	Cnidaria	203	Animalia	0.3770	0.014
During	Mollusca	278	Calanoida	0.3770	0.014
During	Cnidaria	128	*Subeucalanus mucronatus* (v)	0.3770	0.016
During	Copepod 3	126	*Stylocheiron sp.*	0.3770	0.018
During	Mollusca	152	*Subeucalanus mucronatus* (v)	0.3770	0.021
During	Cnidaria	81	Echinodermata	0.3717	0.050
During	Copepod 3	246	Calanoida	0.3565	0.020
During	Cnidaria	201	Eukaryota	0.3565	0.022
During	Cnidaria	73	Unknown	0.3565	0.037
During	Cnidaria	105	Hydrozoa	0.3565	0.037
During	Copepod 2	107	Mollusca	0.3381	0.027
During	Mollusca	211	Arthropoda	0.3381	0.029
During	Mollusca	108	Annelida	0.3381	0.037
During	Crustacea	13	Animalia	0.3381	0.038
During	Cnidaria	46	*Pleurobranchus hilli*	0.3381	0.040
During	Mollusca	135	Caenogastropoda	0.3381	0.043
During	Universal	59	*Creseis sp.*	0.3381	0.044
During	Mollusca	5	*Cliona sp.*	0.3216	0.037
During	Copepod 1	60	Arthropoda	0.3216	0.037
During	Copepod 3	300	*Clausocalanus sp.*	0.3216	0.040
During	Crustacea	67	Eukaryota	0.3216	0.047
During	Universal	87	Chromista	0.3216	0.050
During	Copepod 3	150	Arthropoda	0.3130	0.019
During	Mollusca	121	Mollusca	0.3130	0.026
During	Copepod 3	264	*Euphausia hemigibba*	0.2988	0.033
During	Mollusca	185	Gastropoda	0.2988	0.048
After	Mollusca	4	Eukaryota	0.8780	0.001
After	Mollusca	64	*Oncaea sp.* (Two – 100%)	0.7073	0.001
After	Cnidaria	16	Chlorophyta	0.6681	0.001
After	Cnidaria	5	Prasinophyceae	0.6585	0.001
After	Copepod 3	28	Muricidae	0.6352	0.001
After	Copepod 1	33	Gastropoda	0.6341	0.002
After	Copepod 1	36	Arthropoda	0.5746	0.002
After	Copepod 1	4	*Oncaea waldemari* (v)	0.5463	0.002
After	Cnidaria	104	Chlorophyta	0.5158	0.018
After	Universal	13	Chlorophyta	0.5131	0.002
After	Mollusca	75	Arthropoda	0.5122	0.014
After	Copepod 3	50	*Paracalanus aculeatus*	0.4920	0.016
After	Copepod 1	30	Neogastropoda	0.4878	0.015
After	Copepod 2	25	*Paracalanus sp. (*Two at 100%)	0.4696	0.034
After	Copepod 3	89	Arthropoda	0.4634	0.026
After	Copepod 1	44	Triconia sp.	0.4634	0.029
After	Universal	14	Chlorellaceae (Many at 99%)	0.4445	0.036
After	Mollusca	45	Animalia	0.4390	0.038
After	Mollusca	96	Animalia	0.4146	0.031
After	Copepod 3	107	Calanoida	0.4146	0.032

Heatwave	Assay	OTU	Taxa	Indicator value	*p* value
After	Mollusca	128	Eukaryota	0.4146	0.034
After	Cnidaria	155	Eukaryota	0.4146	0.038
After	Copepod 3	44	Animalia	0.4125	0.016
After	Copepod 3	148	Decapoda	0.3902	0.033
After	Copepod 3	92	Gastropoda	0.3902	0.039
After	Copepod 3	138	Calanoida	0.3902	0.050

(v) Matched to vouchered specimen sequence
