# Supplementary material for: Marine environmental DNA biomonitoring reveals seasonal patterns in biodiversity and identifies ecosystem responses to anomalous climatic events
Source: PLoS Genet. 2019 Feb 8;15(2):e1007943. doi: 10.1371/journal.pgen.1007943 (PMC6368286; doi:10.1371/journal.pgen.1007943)
Supplement: S14 Table — (PDF) [file pgen.1007943.s014.pdf]

Table S14: Indicator species analysis for 17-month heatwave variation—*Indval* [8].

Heatwave	Assay	OTU	Taxa	Indicator value	*p* value
Before	Cnidaria	165	Pythiales	0.7778	0.001
Before	Cnidaria	21	Hydrozoa	0.5234	0.001
Before	Copepod 1	74	*Paracalanus Indicus*	0.4892	0.002
Before	Mollusca	36	*Creseis sp.*	0.4705	0.004
Before	Universal	23	Syndiniales	0.4701	0.004
Before	Cnidaria	22	*Penilia avirostris*	0.4549	0.015
Before	Copepod 1	57	Arthropoda	0.4497	0.001
Before	Cnidaria	54	*Acartia negligens*	0.4460	0.016
Before	Cnidaria	87	Chlorophyta	0.4395	0.014
Before	Cnidaria	175	Animalia	0.4291	0.003
Before	Cnidaria	24	*Aglaura hemistoma*	0.4223	0.012
Before	Copepod 3	27	Clausocalanidae	0.4105	0.041
Before	Mollusca	137	Calanoida	0.4029	0.012
Before	Copepod 2	14	Calanoida	0.3980	0.015
Before	Mollusca	216	Arthropoda	0.3951	0.003
Before	Copepod 2	11	Arthropoda	0.3951	0.003
Before	Cnidaria	58	Arthropoda	0.3836	0.017
Before	Cnidaria	41	*Ophiura kinbergi*	0.3830	0.036
Before	Cnidaria	76	Chlorophyta	0.3652	0.012
Before	Mollusca	333	*Oncaea venusta typica*	0.3568	0.009
Before	Copepod 1	114	Arthropoda	0.3363	0.007
Before	Copepod 2	86	*Ophiura kinbergi*	0.3214	0.031
Before	Copepod 2	113	*Temora discaudata* (v)	0.2750	0.043
Before	Cnidaria	168	Plantae	0.2745	0.013
Before	Copepod 1	5	Mollusca	0.2617	0.030
Before	Mollusca	188	Pyramimonadophyceae	0.2333	0.029
Before	Copepod 1	110	Gastropoda	0.2222	0.025
Before	Mollusca	252	Polychaeta	0.2222	0.028
Before	Copepod 3	285	Calanoida	0.2222	0.029
Before	Copepod 1	133	Gastropoda	0.2222	0.036
Before	Cnidaria	127	Chlorophyta	0.1915	0.040
Before	Cnidaria	93	Leptothecata	0.1915	0.042
During	Copepod 2	35	Paracalanidae	0.4342	0.016
During	Copepod 3	35	Calanoida	0.4264	0.008
During	Copepod 3	130	*Subeucalanus sp.* (Two – 100%(v))	0.4043	0.019
During	Copepod 3	72	*Candacia truncate* (v)	0.3988	0.006
During	Copepod 3	52	*Eucalanus pseudattenuatus* (v)	0.3929	0.022
During	Copepod 3	83	*Acrocalanus gracilis*	0.3840	0.041
During	Mollusca	280	*Undinula vulgaris*	0.3829	0.010
During	Copepod 3	165	*Clausocalanus jobei* (v)	0.3713	0.040
During	Crustacea	31	Animalia	0.3663	0.004
During	Mollusca	17	*Lucifer sp.* (v)	0.3663	0.008
During	Copepod 2	105	*Acrocalanus gracilis* (v)	0.3634	0.010
During	Universal	48	*Subeucalanus pileatus*	0.3496	0.041
During	Copepod 2	107	Mollusca	0.3381	0.029
During	Copepod 1	103	Clausocalanidae	0.3363	0.022
During	Mollusca	115	*Creseis sp.*	0.3352	0.018
During	Universal	71	*Temora sp.*	0.3352	0.027

Heatwave	Assay	OTU	Taxa	Indicator value	*p* value
During	Mollusca	148	Prasinophyceae	0.3349	0.038
During	Copepod 3	121	Sagittidae	0.3245	0.050
During	Copepod 3	111	*Lucifer intermedius*	0.3150	0.010
During	Mollusca	215	Arthropoda	0.3043	0.042
During	Copepod 3	95	Arthropoda	0.3029	0.048
During	Copepod 1	122	*Undinula vulgaris*	0.2844	0.029
During	Crustacea	13	Animalia	0.2778	0.009
During	Universal	87	Chromista	0.2461	0.016
During	Mollusca	65	Decapoda	0.2461	0.026
During	Universal	61	Syndiniales	0.2461	0.028
During	Copepod 3	301	Potamididae	0.2314	0.022
During	Mollusca	179	Hexanauplia	0.2314	0.037
During	Cnidaria	105	Hydrozoa	0.2222	0.020
During	Copepod 2	64	*Candacia catula*	0.2222	0.022
During	Copepod 3	119	Chaetognatha	0.1915	0.046
After	Cnidaria	5	Prasinophyceae	0.7940	0.001
After	Mollusca	4	Eukaryota	0.6350	0.001
After	Cnidaria	16	Chlorophyta	0.6036	0.001
After	Mollusca	64	*Oncaea sp.* (Two – 100%)	0.5843	0.001
After	Copepod 1	4	Oncaea waldemari (v)	0.5455	0.001
After	Universal	13	Chlorophyta	0.4936	0.001
After	Copepod 1	19	Arthropoda	0.4802	0.003
After	Copepod 3	28	Muricidae	0.4603	0.006
After	Copepod 2	29	*Calocalanus styliremis* (v)	0.4474	0.015
After	Cnidaria	11	Hydrozoa	0.4346	0.001
After	Copepod 1	36	Arthropoda	0.4309	0.008
After	Cnidaria	36	Arthropoda	0.4227	0.021
After	Mollusca	27	Calanoida	0.4212	0.038
After	Copepod 1	9	Triconia sp.	0.4208	0.025
After	Copepod 1	17	Arthropoda	0.4133	0.020
After	Cnidaria	104	Chlorophyta	0.4100	0.006
After	Copepod 1	20	Arthropoda	0.4094	0.015
After	Copepod 3	148	Decapoda	0.4091	0.010
After	Copepod 3	44	Animalia	0.4047	0.012
After	Mollusca	30	*Cacozeliana granarium*	0.3971	0.044
After	Copepod 1	7	Arthropoda	0.3945	0.026
After	Copepod 3	86	*Calocalanus styliremis*	0.3821	0.042
After	Copepod 1	84	Arthropoda	0.3746	0.014
After	Copepod 1	54	Arthropoda	0.3701	0.044
After	Copepod 1	42	Arthropoda	0.3609	0.013
After	Copepod 3	190	*Calocalanus sp.*	0.3403	0.018
After	Copepod 1	27	Acartiidae	0.3400	0.040
After	Copepod 1	31	Ataxocerithium	0.3296	0.050
After	Cnidaria	155	Eukaryota	0.3140	0.030
After	Cnidaria	25	Gastropoda	0.3086	0.044
After	Copepod 1	47	Arthropoda	0.2857	0.024
After	Copepod 3	315	Calanoida	0.2724	0.035
After	Mollusca	309	Mollusca	0.2500	0.028
