# Supplementary material for: Marine environmental DNA biomonitoring reveals seasonal patterns in biodiversity and identifies ecosystem responses to anomalous climatic events
Source: PLoS Genet. 2019 Feb 8;15(2):e1007943. doi: 10.1371/journal.pgen.1007943 (PMC6368286; doi:10.1371/journal.pgen.1007943)
Supplement: S15 Table — (PDF) [file pgen.1007943.s015.pdf]

Table S15: Alternative linear models for Assemblage and Richness.

Assay used	OTU diversity test	Most parsimonious model (R^2)	Alternative Model 1	Alternative Model 2	Alternative Model 3	Alternative Model 4	Alternative Model 5
Cnidaria	Assemblage	SST, Salinity, Silicate (0.162)	Silicate	SST, Silicate	SST, Salinity, Silicate, Phosphate	SST, Salinity, Silicate, Nitrate, Phosphate	SST, Salinity, Silicate, Nitrate, Phosphate, Ammonium
	AIC	404.45	405.92	405.12	405.02	405.51	406.10
	Richness	SST, Silicate (0.112)	Silicate	SST, Silicate, Phosphate	SST, Silicate, Phosphate, Ammonium		
	AIC	447.24	447.30	447.41	449.05		
Copepod 1	Assemblage	SST, Salinity, Silicate (0.155)	Silicate	SST, Salinity, Silicate, Nitrate, Phosphate, Ammonium	SST, Salinity, Silicate, Nitrate, Phosphate, Ammonium	SST, Salinity, Silicate, Nitrate, Phosphate, Ammonium	
	AIC	404.62	405.3	405.02	405.33	406.17	
	Richness	Salinity (0.067)	Salinity, Phosphate				
	AIC	451.13	452.61				
Copepod 2	Assemblage	SST, Salinity, Silicate (0.230)	Salinity, Silicate	SST, Salinity, Silicate, Ammonium			
	AIC	382.78	383.94	383.59			
	Richness	Salinity, Silicate (0.309)	Salinity, Silicate, Ammonium	Salinity, Silicate, Phosphate, Ammonium			
	AIC	427.16	427.60	429.03			
Copepod 3	Assemblage	SST, Salinity, Silicate (0.227)	Salinity	SST, Salinity	SST, Salinity, Silicate, Ammonium	SST, Salinity, Silicate, Phosphate, Ammonium	
	AIC	383.36	385.25	383.83	383.79	384.49	
	Richness	SST, Salinity, Ammonium (0.392)	SST, Salinity	SST, Salinity, Phosphate, Ammonium			
	AIC	492.79	492.90	494.31			
Crustacea	Assemblage	SST, Salinity (0.098)	Silicate	SST, Salinity, Ammonium	SST, Salinity, Silicate, Ammonium	SST, Salinity, Silicate, Phosphate, Ammonium	

Assay used	OTU diversity test	Most parsimonious model (R^2)	Alternative Model 1	Alternative Model 2	Alternative Model 3	Alternative Model 4	Alternative Model 5
	AIC	405.52	405.97	405.89	406.61	407.39	
	Richness	SST, Ammonium (0.183)	SST, Salinity, Ammonium	SST, Salinity, Silicate, Ammonium			
	AIC	376.85	377.39	377.43			
Fish	Assemblage	SST, Salinity, Ammonium (0.147)	Salinity	SST, Salinity	SST, Salinity, Phosphate, Ammonium	SST, Salinity, Silicate, Nitrate, Phosphate	SST, Salinity, Silicate, Nitrate, Phosphate, Ammonium
	AIC	427.43	428.33	427.77	428.07	428.25	428.9
	Richness	Nitrate, Salinity, Silicate, Phosphate (0.251)	Nitrate, Silicate	Nitrate, Salinity, Silicate	SST, Nitrate, Salinity, Silicate, Phosphate		
	AIC	262.88	263.95	263.88	264.82		
Mollusca	Assemblage	SST, Salinity, Silicate (0.197)	SST, Salinity	SST, Salinity, Silicate, Ammonium	SST, Salinity, Silicate, Phosphate, Ammonium	SST, Salinity, Silicate, Nitrate, Phosphate, Ammonium	
	AIC	388.46	389.22	388.85	389.25	390.17	
	Richness	SST (0.061)	SST, Ammonium	SST, Silicate, Ammonium			
	AIC	499.91	500.94	501.65			
Universal	Assemblage	SST, Salinity, Silicate (0.140)	Salinity	Salinity, Silicate	SST, Salinity, Silicate, Phosphate	SST, Salinity, Silicate, Phosphate, Ammonium	SST, Salinity, Silicate, Nitrate, Phosphate, Ammonium
	AIC	371.36	372.12	371.4	370.95	370.95	371.89
	Richness	SST, Salinity, Phosphate (0.212)	SST, Nitrate, Salinity, Phosphate				
	AIC	374.71	376.55				
