# Supplementary material for: Marine environmental DNA biomonitoring reveals seasonal patterns in biodiversity and identifies ecosystem responses to anomalous climatic events
Source: PLoS Genet. 2019 Feb 8;15(2):e1007943. doi: 10.1371/journal.pgen.1007943 (PMC6368286; doi:10.1371/journal.pgen.1007943)
Supplement: S16 Table — (PDF) [file pgen.1007943.s016.pdf]

Table S16: Metabarcoding PCR assays developed and tested for this study.

PCR assay	Primer set used	Target Taxa	Gene	Primer sequence	Amplicon length (bp)	Reference	Assay T_a (°C)	Mostly detected
Copepod 3	Acartia F<br>Acartia R	Calanoida	COI	5′ GGRGAYGATCARRTYTATAAYGT 3′<br>5′ TTYATWCGWGGAAAHGCYATRTC 3′	~103	This study	52	Malacostraca, Hexanauplia, Actinopterygii
Acrocalanus	Acrocalanus F<br>Acrocalanus R	Calanoida	COI	5′ CTGTTTAYCCWCCTCTTTCTAG 3′<br>5′ TCTARTAAYATDCCAAATACTCGT 3′	~111	Pilot study	50	Hexanauplia
Branchiostoma	Branchiostoma F<br>Branchiostoma R	Leptocardii	COI	5′ CRGAYATRGCKTTYCCYCG 3′<br>5′ AAAATTGAYGAYACMCCWGC 3′	~169	Pilot study	50	Mollusca
Calanus	Calanus F<br>Calanus R	Calanoida	COI	5′ GCWTCYGTNGAYTTYGCWAT 3′<br>5′ CCSCCCACATCAYAMAATG 3′	~212	Pilot study	51	Very little
Calocalanus	Calocalanus F<br>Calocalanus R	Calanoida	COI	5′ CTGGDTCNTTRATTGGRGATG 3′<br>5′ GAHAAAGGDGGRTAAACWGTTC 3′	~225	Pilot study	51	Hexanauplia
Candacia	Candacia F<br>Candacia R	Calanoida	COI	5′ GGVRSATSAGTDGACTTTGC 3′<br>5′ GCAAANAGAGGYATWCGNTC 3′	~97	Pilot study	51	Hexanauplia
Cnidaria	Cnidaria F<br>Cnidaria R	Cnidarians	COI	5′ CATGATHTTYTTCWTDGTMATGCC 3′<br>5′ GTYCAWCCWGTWCCWRCYCC 3′	~145	This study	52	Cnidaria, Echinodermata
Copilia	Copilia F<br>Copilia R	Cyclopoida	COI	5′ CGCAATCTTCTCTCTACACC 3′<br>5′ GGTAATGAGAGGAGAAGTAGG 3′	~124	Pilot study	53	*Copilia mirabilis*
Creseis	Creseis F<br>Creseis R	Gastropoda	COI	5′ CATTCTGACTHCTHCCTCCWTC 3′<br>5′ GCMCCHAGAATAGAAGAAATACC 3′	~136	Pilot study	53	Mollusca
Crust 16S	Crust16S_F(short)<br>Crust16S_R(short)	Crustaceans	16S rRNA	5′ GGGACGATAAGACCCTATA 3′<br>5′ ATTACGCTGTTATCCCTAAAG 3′	~170	Berry et al. [1]	51	Malacostraca
Cyttarocylis	Cyttarocylis F<br>Cyttarocylis R	Ciliophora	18S rRNA	5′ CGCGTAAATTACCCAATCCTG 3′<br>5′ CTAACAGAAATCCAACTACGAGC 3′	~169	Pilot study	56	Chromis
Doliolum	Doliolum F<br>Doliolum R	Thaliacea	18S rRNA	5′ GGGATGCGTGCTTTTATC 3′<br>5′ CTTGGATGTGGTAGCCG 3′	~196	Pilot study	52	Very little
Eucalanus	Eucalanus F<br>Eucalanus R	Calanoida	COI	5′ GTAGARAGAGGNGCHGGWAC 3′<br>5′ GCAGTAATVACKACDGCYCA 3′	~184	Pilot study	52	Hexanauplia

PCR assay	Primer set used	Target Taxa	Gene	Primer sequence	Amplicon length (bp)	Reference	Assay T_a (°C)	Mostly detected
Fish 16S	Fish16sF/D<br>16s2R<br>(degenerate)	Fish	16S rRNA	5′ GACCCTATGGAGCTTTAGAC 3′<br>5′ CGCTGTTATCCCTADRGTAACT 3′	~200	F- Berry et al. [1]<br>R-Deagle et al. [2]	54	Actinopterygii
Mollusca	Limacina F<br>Limacina R	Mollusca	COI	5′ TAATTGGNGGVTTTGGRAAYTG 3′<br>5′ GTTCAHCCTRAYCCTRCNCC 3′	~118	This study	52	Hexanauplia, Cnidaria, Mollusca
Copepod 2	Lucicutia F<br>Lucicutia R	Calanoida	COI	5′ CCHGAYATAGCTTTYCCHCG 3′<br>5′ GAAAAAATTGCAAAATCTACDGATC 3′	~134	This study	52	Hexanauplia, Mollusca
Lucifer	Lucifer F<br>Lucifer R	Decapoda	COI	5′ GGAGAYGAYCARATTTATAATGTAG 3′<br>5′ GAGGRAAWGCYATATCAGGAG 3′	~96	Pilot study	51	Hexanauplia
Oithona	Oithona F<br>Oithona R	Cyclopoida	COI	5′ CCAGATATAGCHTTYCCHCG 3′<br>5′ GAAGAHACHCCYGCYAARTG 3′	~163	Pilot study	52	Hexanauplia
Oncaea	Oncaea F<br>Oncaea R	Cyclopoida	COI	5′ TTGGSKGKTTYGGWAATTG 3′<br>5′ GCTAARTGAAGWSHAAAAATRG 3′	~194	Pilot study	48	Hexanauplia
Paracalanus	Paracalanus F<br>Paracalanus R	Calanoida	COI	5′ CCRTTAATAYTAGGAGCAGC 3′<br>5′ CTAAAATWGAACTWACWCCTGC 3′	~187	Pilot study	51	Hexanauplia
Pontellidae	Pontellidae F<br>Pontellidae R	Calanoida	COI	5′ GRGCAGGWACDGGDTGRAC 3′<br>5′ CGATCYAAAATTATYCCAAAHACWCG 3′	~133	Pilot study	54	Hexanauplia
S_Ceph 16S	S_Cephalopoda_F<br>S_Cephalopoda_R	Cephalopoda	16S rRNA	5′ GCTRGAATGAATGGTTTGAC 3′<br>5′ TCAWTAGGGTCTTCTCGTCC 3′	~70	Peters et al. [11]	50	Mollusca<br>Crustacea
Copepod 1	Triconia F<br>Triconia R	Cyclopoida	COI	5′ CAGGVTCTTTAYTWGGRGATG 3′<br>5′ AAAATCTTATATTATTTARBCGRGG 3′	~131	This study	49	Hexanauplia, Mollusca
Urchin	Urchin F<br>Urchin R	Echinodermata	16S rRNA	5′ GACGAGAAGACCCTGDCG 3′<br>5′ CGCTGTTATCCCTRCGGTAAC 3′	~200-230	Pilot study	55	Echinodermata
WSP	WSP F<br>WSP R	Decapoda	COI	5′ AGWYTWATTATYCGRGCWGA 3′<br>5′ TCARTTWCCAAATCCWCC 3′	~107	Pilot study	48	Malacostraca

"F" refers to the forward primer; "R" refers to the reverse primer.
