# Supplementary material for: Marine environmental DNA biomonitoring reveals seasonal patterns in biodiversity and identifies ecosystem responses to anomalous climatic events
Source: PLoS Genet. 2019 Feb 8;15(2):e1007943. doi: 10.1371/journal.pgen.1007943 (PMC6368286; doi:10.1371/journal.pgen.1007943)
Supplement: S3 Fig — The 2011 and 2012 heatwave events produce the two highest peaks—Extracted from the Bureau of Meteorology time series graphs [9]. (PDF) [file pgen.1007943.s019.pdf]

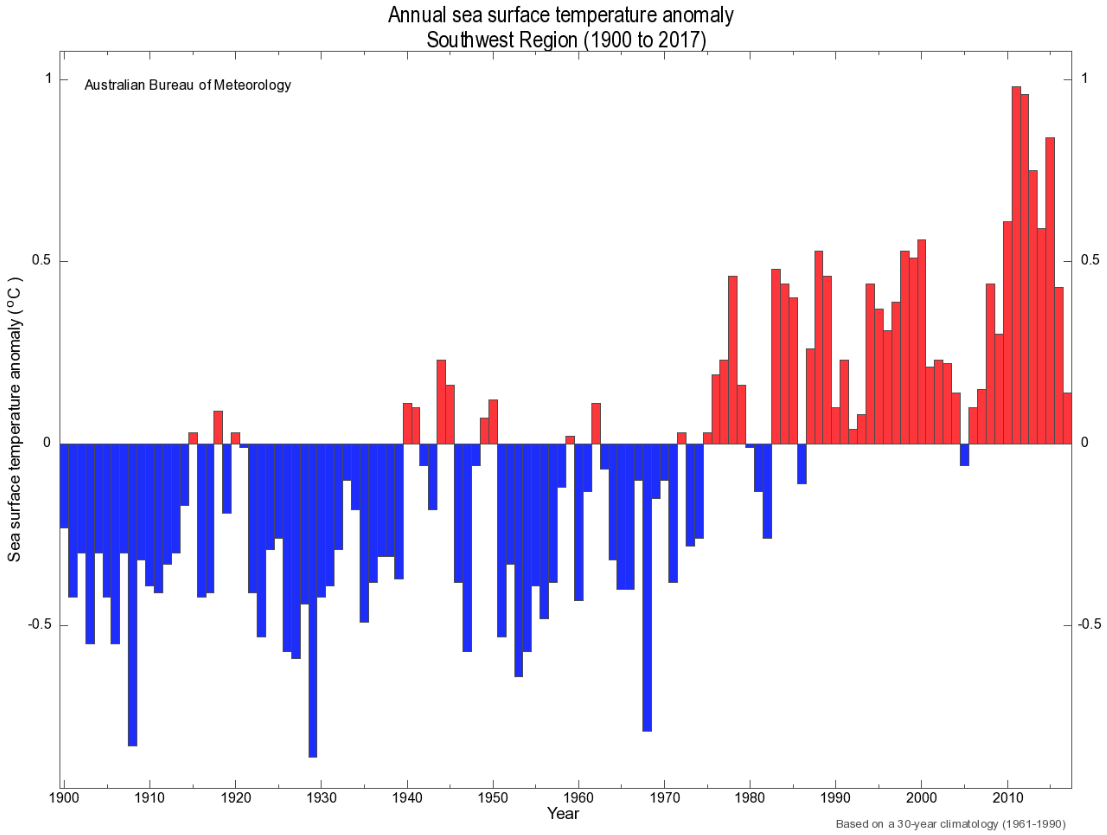

Figure S3: Annual sea surface temperature anomalies from 1900-2017. The 2011 and 2012 heatwave events produce the two highest peaks —Extracted from the Bureau of Meteorology time series graphs [9].
