# Supplementary material for: Marine environmental DNA biomonitoring reveals seasonal patterns in biodiversity and identifies ecosystem responses to anomalous climatic events
Source: PLoS Genet. 2019 Feb 8;15(2):e1007943. doi: 10.1371/journal.pgen.1007943 (PMC6368286; doi:10.1371/journal.pgen.1007943)
Supplement: S1 Text — (PDF) [file pgen.1007943.s021.pdf]

References – Supplementary information

1. Berry TE, Osterrieder SK, Murray DC, Coghlan ML, Richardson AJ, Grealy AK, et al. DNA metabarcoding for diet analysis and biodiversity: A case study using the endangered Australian sea lion (*Neophoca cinerea*). Ecol Evol. 2017;7(14):5435-53.

2. Deagle BE, Gales NJ, Evans K, Jarman SN, Robinson S, Trebilco R, et al. Studying Seabird Diet through Genetic Analysis of Faeces: A Case Study on Macaroni Penguins (*Eudyptes chrysolophus*). PLoS ONE. 2007;2(9):e831.

3. Pochon X, Bott NJ, Smith KF, Wood SA. Evaluating Detection Limits of Next-Generation Sequencing for the Surveillance and Monitoring of International Marine Pests. PLOS ONE. 2013;8(9):e73935.

4. Davies Claire H, Armstrong Amelia J, Baird M, Coman F, Edgar S, Gaughan D, et al. Over 75 years of zooplankton data from Australia. Ecology. 2014;95(11):3229-.

5. ALA. Atlas of Living Australia website http://www.ala.org.au2016

6. Thompson H. Pelagic tunicates of Australia. 1948.

7. Anderson M, Gorley RN, Clarke RK. Permanova+ for Primer: Guide to Software and Statisticl Methods: Primer-E Limited; 2008.

8. Roberts DW. labdsv: Ordination and Multivariate Analysis for Ecology. 2016;R package version 1.8-0.

9. Australian Bureau of Meteorology. Australian climate variability & change - Time series graphs at: http://www.bom.gov.au/climate/change/ (data accessed in December 2018).

10. Kearse M, Moir R, Wilson A, Stones-Havas S, Cheung M, Sturrock S, et al. Geneious Basic: An integrated and extendable desktop software platform for the organization and analysis of sequence data. Bioinformatics. 2012;28(12):1647-9.

11. Peters KJ, Ophelkeller K, Bott NJ, Deagle BE, Jarman SN, Goldsworthy SD. Fine-scale diet of the Australian sea lion (*Neophoca cinerea*) using DNA-based analysis of faeces. Marine Ecology. 2014:n/a-n/a.
